# A two-stage computational approach to predict novel ligands for a chemosensory receptor

**DOI:** 10.1016/j.crstbi.2020.10.001

**Published:** 2020-10-09

**Authors:** Amara Jabeen, Ramya Vijayram, Shoba Ranganathan

**Affiliations:** a Department of Molecular Sciences, Macquarie University, Sydney, NSW 2109, Australia; b Department of Biotechnology, Bhupat and Jyoti Mehta School of Biosciences, Indian Institute of Technology Madras, Chennai 600036, Tamilnadu, India

**Keywords:** Olfactory receptor, OR1A2, Molecular dynamics, Atomic property field, Virtual ligand screening, Binding free energy calculation, Hydrophobicity correspondence, Amber, Assisted model Building with Energy Refinement, APF, Atomic property field, CSF, Cerebrospinal fluid, ECL, Extracellular loop, GPCR, G protein coupled receptor, HCMV, Human cytomegalovirus, HMDB, Human metabolome database, LBVS, Ligand based virtual screening, LC, Lung carcinoids, MD, Molecular dynamics, MMGBSA, Molecular mechanics generalized born surface area, MMPBSA, Molecular mechanics Poisson–Boltzmann surface area, NAFLD, Non-alcoholic fatty liver disease, NASH, Nonalcoholic steatohepatitis, OR, olfactory receptor, PMEMD, Particle-Mesh Ewald Molecular Dynamics, POPC, 1-palmitoyl-2-oleoyl-sn-glycero- 3-phosphatidylcholine, RMSD, Root mean square deviation, RMSF, Root mean square fluctuation, SBVS, Structure based virtual screening, SSD, Sum of squared difference, TM, Transmembrane

## Abstract

Olfactory receptor (OR) 1A2 is the member of largest superfamily of G protein-coupled receptors (GPCRs). OR1A2 is an ectopically expressed receptor with only 13 known ligands, implicated in reducing hepatocellular carcinoma progression, with enormous therapeutic potential. We have developed a two-stage screening approach to identify novel putative ligands of OR1A2. We first used a pharmacophore model based on atomic property field (APF) to virtually screen a library of 5942 human metabolites. We then carried out structure-based virtual screening (SBVS) for predicting the potential agonists, based on a 3D homology model of OR1A2. This model was developed using a biophysical approach for template selection, based on multiple parameters including hydrophobicity correspondence, applied to the complete set of available GPCR structures to pick the most appropriate template. Finally, the membrane-embedded 3D model was refined by molecular dynamics (MD) simulations in both the *apo* and *holo* forms. The refined model in the *apo* form was selected for SBVS. Four novel small molecules were identified as strong binders to this olfactory receptor on the basis of computed binding energies.

## Highlights

• A two-stage screening has led to the prediction of structurally similar ligands for OR1A2.
• A biophysical approach, including a novel parameter of hydrophobicity correspondence, is presented for template selection.
• Available GPCR templates for OR1A2 have been comprehensively compared using our proposed biophysical approach.
• 1U19 is suggested to be the best template for OR1A2 on the basis of biophysical properties.
• Combining ligand-based and structure-based screening might facilitate decoding of ORs molecular receptive code.

## Graphical abstract

**Figure:**
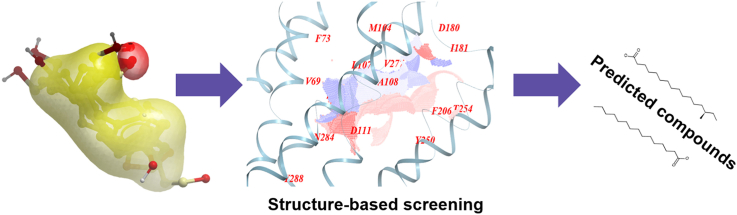

## 1 Introduction

Our sense of olfaction is a consequence of chemosensory events led by interaction of 405 olfactory receptors (ORs) with numerous odorants, in a combinatorial manner (Trimmer and Mainland, 2017). ORs are the largest superfamily of the seven transmembrane (TM) domain G protein-coupled receptors (GPCRs), with 18 families and 300 subfamilies. Similar to non-olfactory GPCRs, the ORs signalling cascade is initiated once odorants bind to inactive ORs. Upon odorant binding, a specific G protein is activated, which then initiates the olfactory signal transduction cascade (Buck and Axel, 1991). Besides roles in olfaction, ORs are now known to be involved in wide variety of chemosensory processes, especially in non-olfactory, i.e. ectopic tissues. However, they are not fully functionally characterized as yet in the majority of tissues, owing to difficulties associated with their heterologous functional expression (Tsai et al., 2017). Also, the evidence for their ectopic expression is at the mRNA level and not at the protein level in several tissues (Jabeen et al., 2019a). Among their diversified functions in the human body are mediation of rennin secretion and blood pressure, negative chronotropic effect on fetal and adult heart, serotonin release within gut enterochromaffin cells, inducing melanocyte pigmentation and differentiation, and sperm migration (Dalesio et al., 2018).

Besides exhibiting important functions within healthy tissues, ORs are known to be associated with pathophysiological conditions. OR7C1 maintains colon cancer initiating cells, OR51B5 leads to the inhibition of cell proliferation in K562 cells, OR51E1 acts as a tumour biomarker for lung carcinoids (LC) in somatostatin receptor-negative tumour patients, OR2J3 activation by helional induces *apo*ptosis and inhibits cell proliferation in non-small-cell lung cancer, OR51E2 acts as a biomarker for prostate cancer (Dalesio et al., 2018) and OR10H1 is the potential biomarker for urinary bladder cancer (Weber et al., 2018a). Recently, 968 cancer cell lines were investigated for the expression of 301 selected OR genes with 49% showing expression of at least one OR (Ranzani et al., 2017). Moreover, OR14I1 is proposed to be the receptor for human cytomegalovirus (HCMV) infection (Xiaofei et al., 2019). Therefore, ORs might be the potential targets for the pharmaceutical industry. Following clinical trials, a ligand for OR2AT4, Sandalore®, is now regarded as a therapeutic alternative to treat hair loss (Jimenez et al., 2020). Sandalore® as well as another OR2AT4 ligand, Brahmanol have wound-healing properties (Busse et al., 2014). Also, Santalol and Sandranol are OR10H1 ligands, that are used in German clinics to treat bladder cancer although the compounds are not clinically tested yet (Weber et al., 2018a). Preclinical studies are currently under way on Azelaic acid, a ligand for the mouse OLFR544, for obesity and subcutaneous fat reduction (Lee et al., 2019).

Defining the odorant-receptor pairs is critical for understanding smell perception and for functionally characterizing ectopically expressed ORs in non-nasal tissues (Weber et al., 2018a). Given the vast array of odorant molecules, the ~400 human ORs and combinatorial coding complexity, it is a challenging task to define receptor-odorant pairs (Bavan et al., 2014). Difficulties in heterologous expression of ORs and current unavailability of experimentally resolved 3D structure of any OR have further hampered our understanding of molecular mechanism underlying physiological and pathophysiological roles of ORs (Jabeen et al., 2019a). Only four members of the ORs are known as proteins, with the remaining ORs regarded as “missing proteins” since they lack significant proteomics evidence (Baker et al., 2017). 107 ORs have some kind of orthogonal evidence including deorphanization, with 87 ORs having known ligands (Jabeen et al., 2019a). Of the deorphanized ORs, six (OR1A1, OR1G1, OR2W1, OR51E1 OR51E2, OR52D1) have sufficient known agonists and are suitable for the application of machine learning (ML), in order to further extend their chemical space. ML has already been applied to OR1G1 (Jabeen and Ranganathan, 2019), OR1A1, OR2W1 and OR51E1 (Bushdid et al., 2018). The remaining deorphanized ORs have limited agonist data and are not suitable for ML. For these ORs, a ligand-based pharmacophore method coupled with structure-based virtual screening (SBVS) is the preferred approach. Homology modelling can be used for SBVS in the absence of experimental structures for ORs. However, there are several challenges associated with modelling ORs. Firstly, ORs show low sequence identity (<30%) with the currently available experimental GPCR structures (Alfonso-Prieto et al., 2019), although more than 18% non-olfactory GPCRs can be modelled with a 35% sequence identity cutoff for homology modelling (Stevens et al., 2013). Secondly, although ORs share some common motifs with other GPCRs in almost every TM, the CWxP motif in TM6 which is known as a toggle switch for GPCRs and an ionic block between TM3 and TM6 are absent in all ORs (de March et al., 2015a), confounding the alignment of OR TM regions with template structures.

To identify novel ligands for ORs with limited experimental ligand profile, structure-based virtual screening (SBVS) using a homology model of OR51E2, based on the structure of the human adrenergic beta-2-receptor (β2-AR), has been used to discover novel agonists (Abaffy et al., 2018). Recent *in silico* approaches coupled with *in vitro* assays have elucidated the ligand-binding cradle for different ORs (Geithe et al., 2017; Ahmed et al., 2018; Wolf et al., 2017) and contributed to our understanding of the OR activation mechanism (de March et al., 2018). Multiple templates, such as human M2 muscarinic receptor (Stevens et al., 2013), β2-adrenergic receptor (Abaffy et al., 2018), and bovine rhodopsin (de March et al., 2015a) and (Geithe et al., 2017), have also been used to model ORs. The VS performance has greatly been impacted by template selection (Rataj et al., 2014), specifically when sequence identity is extremely low. It is therefore critical to consider additional parameters for appropriate template selection in order to build reliable homology models.

OR1A2 (UniProtID: Q9Y585) is an OR, known to be ectopically expressed in several tissues at transcript level including blood, brain, heart, liver, pancreas among others as indicated by GeneCards (GCID: GC17P003197) (Stelzer et al., 2016). OR1A2 has been heterologously expressed and identified at the protein level in Huh7 cells, a model system for hepatocellular carcinoma, where the activation of OR1A2 by (*S*)-(−)-citronellal leads to calcium signalling and reduction in cell proliferation (Massberg et al., 2015). Currently, the known odorant space of OR1A2 is comprised primarily of 13 ligands (terpenes, alcohols and aldehydes; Supporting information: Table S1), which are all agonists.

**Fig. 1:**
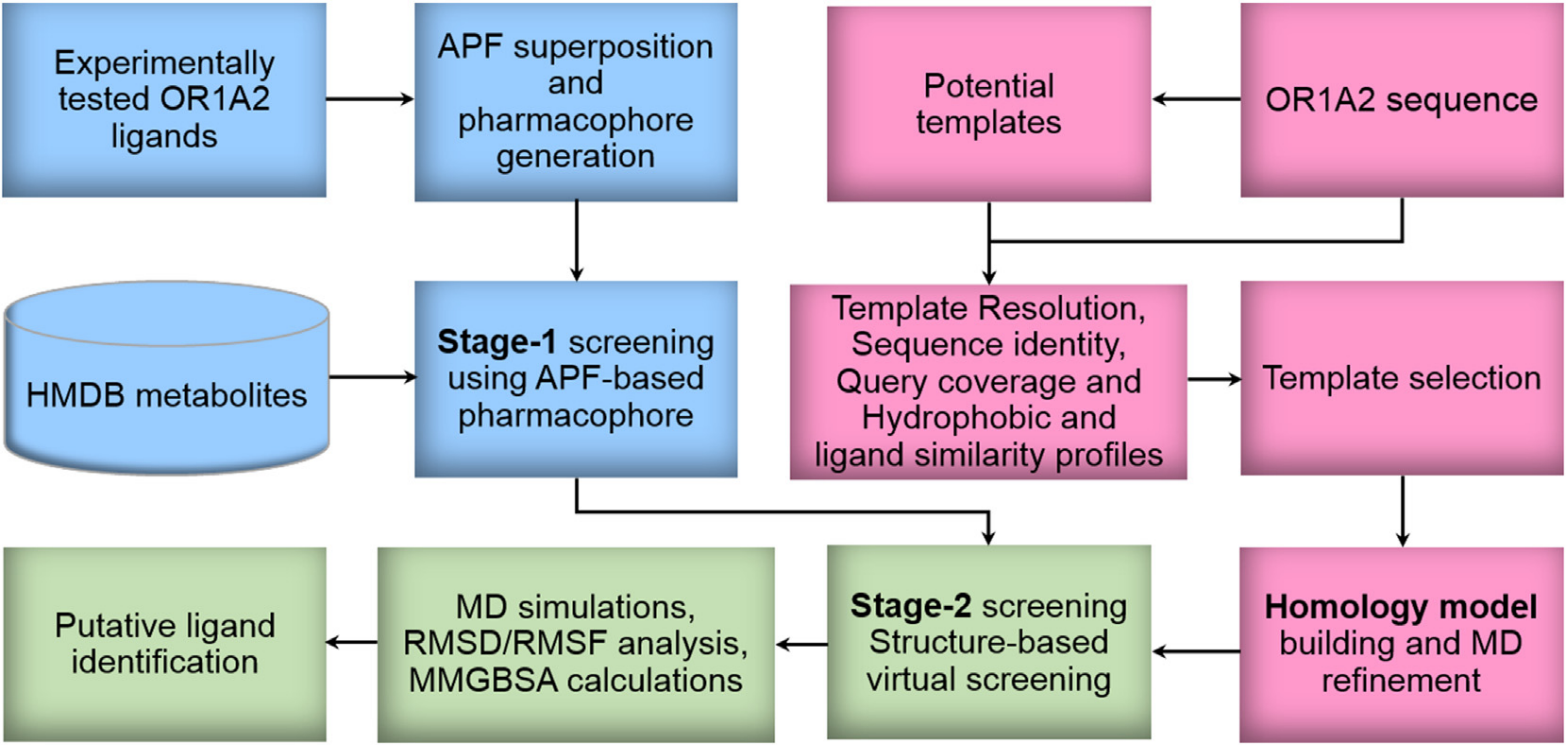
Workflow of integrated virtual ligand screening for OR1A2.

In the current study, we have carried out *in silico* screening of a dataset of 5942 metabolites from serum, bile, urine, saliva, feces, and cerebrospinal fluid (CSF) from the human metabolome database (HMDB) (Wishart et al., 2018), against OR1A2 using a two-stage virtual screening approach, illustrated in Fig. 1. During the first stage, we have selected potential ligands most similar to the known OR1A2 ligands, using a 3D pharmacophore-based atomic property field (APF) superposition (Weber et al., 2018b) approach. In the second stage, we have used structure-based virtual screening (SBVS) using a homology model of OR1A2 to select the metabolites obtained through APF screening. Our earlier homology modelling approach for GPCRs (Jabeen et al., 2019b) has been extended to ORs in the current study, to select the bovine rhodopsin template, rigorously identified by a biophysical approach proposed here, based on multiple parameters including sequence identity, query coverage, resolution, hydrophobicity, ligand profile, and binding site comparison. The top five ligands from SBVS were subjected to molecular dynamics (MD) simulations, with four putative ligands identified, with greater binding affinity than the control ligand.

## 2 Methods

### 2.1 Data acquisition, atomic property field superposition and stage 1 screening

The total 13 experimentally known ligands (Supporting information: Table S1) for OR1A2 reported in the literature (Massberg et al., 2015; Schmiedeberg et al., 2007) were downloaded from PubChem (Kim et al., 2016). We utilized the APF technique, incorporated in the ICM software (MolSoft v.3.8-5, LLC) (Abagyan et al., 1994), for Stage 1 screening. APF is a 3D pharmacophoric potential which is implemented as a grid. Through APF superposition common features of active compounds can be well utilized enough to generate a 3D pharmacophore. APF superposition consider continuous property distribution and uses atomic properties vectors that can be compared in more flexible manner. Seven properties are used by the APF method for superposition, comprising hydrogen bond donors, hydrogen bond acceptors, sp2 hybridization, lipophilicity, size, electropositivity/negativity and charge (Totrov, 2008).

(S)- (−)-citronellal was selected as the ligand template for the superposition based on APF, as it has been reported to be more potent agonist as compared to the other ligands (Schmiedeberg et al., 2007). The rest of the known agonists were flexibly super-positioned to the template using APF, with the effort parameter set to 3 and the ring sampling enabled. A consensus pharmacophore was then developed using the APF-based super-positioned agonists. A total of 5942 serum, bile, urine, saliva, feces, and CSF metabolites were downloaded from HMDB (Wishart et al., 2007) and indexed according to ICM format. The first-stage screening was carried out on the basis of APF superposition. All the ligands with an APF score ≥100 were retrieved. The APF score is based on pseudo-energy computed as mentioned in Eq. (1):

(1) $$E_{APF}=-\sum_{i}\phi_{i}^{j}\;P_{i}\;\left(r^{j}\right)$$

where *ϕ*^*j*^_*i*_ is the property vector with *j* representing atom located at *r*^*j*^ and *P*_*i*_*(****r****)* is the 3D property potential with *i* representing the components.

### 2.2 Biophysical approach for template selection on the basis of hydrophobicity profile

For the second stage SBVS, OR1A2 has no experimental structure, requiring a structural model to be generated. We propose a multi-step biophysical approach for appropriate OR template selection. In the first step, high quality GPCR X-ray structures (resolution ≤ 2.5 Å) were shortlisted. Secondly, hydrophobicity profile and sequence identity between the target and each template were computed. For the set of selected templates from the second step, query coverage between the template and the target was calculated and the best templates identified. Subsequently, the ligand profile of the selected templates from step 3 and the target were compared. In the last step, ligand-binding similarity was calculated between the target and candidate templates. Also, the models were generated with each candidate template, shortlisted at step 3, and the predicted binding sites (PBS) compared by mapping available mutagenesis data.

The hydrophobicity profiles for each helix of the candidate templates and target were generated using Eisenberg scale. Each transmembrane (TM) sequence of the template and target were aligned by tethering center residues together. A moving window approach was used; wherein the average value over all the residues in a window is taken and ascribed to the center residue of the window. For the Eisenberg scale, the window size was set to 11 for identification of putative transmembrane α-helices (Wallace et al., 2004). For each alignment, we have computed the difference in hydrophobicity per residue, using the sum of squared difference (SSD) (Eqs. (2), (3)):

(2) $$H_{n}=\;\sum_{i=n-5}^{n+5}h_{i/11}$$

(3) $$SSD=\sqrt{\sum_{n=1}^{N}{\left(H_{template,n}-H_{target,n}\right)}^{2}}$$

where, *H*_n_ is the calculated hydrophobicity for the aligned template/target residue in the *n*th position of the alignment and *h*_i_ is the hydrophobicity of the *i*th residue from the Eisenberg scale. This value is normalized by dividing it by the number of residues, to evaluate the best template, as the SSD value is dependent on the number of residues in the helix and will not be reflective of the best fit unless a per-residue value is considered.

The ligand profile for the selected templates and the known ligands for OR1A2 was generated sing PubChem fingerprints. The PubChem fingerprints were computed using Knime (Berthold et al., 2009) for chemical similarity comparison. The Tanimoto score was used as a similarity measure.

### 2.3 Homology modelling

The homology models for human OR1A2 was built using Modeller 9.18 (Webb and Sali, 2017). Single and multiple template models were generated, based on the selected templates, with sequence alignment manually adjusted using MEGA7 (Kumar et al., 2016) by aligning centre residues, class A GPCR conserved motifs and cysteine residues forming a disulphide bridge (Wolf et al., 2017). Predicted transmembrane regions were taken from the GRoSS sequence alignment of all known GPCRs sequences (Cvicek et al., 2016). The ligand of each template was initially copied to the OR1A2 model and removed later to create an empty binding pocket within OR1A2. 50 models were generated for each template and with multiple templates. The models with lowest Modeller objective function was selected for further analysis.

### 2.4 Model refinement

We first performed the local refinement within the predicted binding pocket of the generated models. We docked three potent agonists, Helional, Octanal, and (*S*)- (−)-citronellal one by one into the predicted binding pocket and performed the local refinement through induced-fit docking using ICM. For overall refinement, we performed MD simulations of the (*S*)-(−)-citronellal bound models as well as *apo* models. Since OR1A2 is the membrane receptor, the built models were inserted into the membrane for refinement by MD simulations. The initial orientation of OR1A2 model in the membrane was taken from the PPM server (Lomize et al., 2012). The homology model was inserted into a lipid bilayer membrane (of 128 1-palmitoyl-2-oleoyl-sn-glycero-3-phosphatidylcholine (POPC) molecules), solvated with 0.15 M NaCl *via* the CHARMM-GUI server (Jo et al., 2008). For the *apo* model, the total molecular system comprised of 55093 atoms including 128 POPC molecules, 29 Na^+^ ions, 36 Cl^-^ ions and 10,994 water molecules. The Monte Carlo method was used to place the ions and a CHARMM36 force field was assigned to the membrane system. The membrane was equilibrated at constant pressure and temperature (NPT ensemble; 303.15K). The molecular system for the *holo* model was composed of total 54574 atoms including 128 POPC molecules, 28 Na^+^ ions, 35 Cl^-^ ions and 10,812 water molecules.

The lipid bilayer embedded model was subjected to MD model refinement with local structural improvements. The MD simulations were carried out through Assisted Model Building with Energy Refinement (Zhou et al., 2019) 16 package (Case et al., 2016), with the ff14SB force field (Maier et al., 2015) for the receptor and the lipid14 force field (Dickson et al., 2014) for the lipid bilayer. The membrane-embedded OR1A2 model was inserted into a rectangular box and the TIP3P water model (Jorgensen et al., 1983) was employed for solvating the system. Three subsequent steps of energy minimization process were performed during 7,000 iterations. During each minimization step the algorithm was switched from steepest descent to conjugate gradient after 1000 iterations, with restraints on the receptor atomic coordinates of a force constant of 10 kcal mol^−1^Å^2^, for the first two minimization steps alone. No restraints were imposed on the 3rd minimization step. The system temperature was then gradually raised to 310.15 K in three steps (100, 210.15, 310.15) of heating for 205 ps, with restraints applied on the receptor and the lipid part of the membrane. The heating step was followed by 6 ns system equilibration. Production phase was simulated at 310.5 K to yield 40 ns trajectory recorded at each ns. GPU-accelerated Particle-Mesh Ewald Molecular Dynamics (PMEMD) was used wherever periodic boundary conditions were applied.

### 2.5 Stage 2 screening

The refined OR1A2 model was used for Stage 2 screening based on molecular docking. Molecular docking was performed with ICM software. The binding pocket was predicted though ICMPocketFinder (An et al., 2005) and selected on the basis of available mutagenesis data of ligand-binding residues for OR1A2 (Schmiedeberg et al., 2007).

The ligand's covalent geometry was relaxed and flexible ring sampling level was set to 2. Charges were auto-assigned by ICM. Ten conformations were generated for each ligand. The conformation within the vicinity of binding pocket and having the lowest ICM score was selected. The ICM score is calculated as per Eq. (4):

(4) $$\Delta G=\Delta E_{IntFF}+T\Delta S_{Tor}+\alpha_{1}\Delta E_{HBond}+\alpha_{2}\Delta E_{HBDesol}+\alpha_{2}\Delta E_{So1E1}+\alpha_{4}\Delta E_{HPhob}+\alpha_{5}Q_{Size}$$

where *Δ*E_IntFF_ is change in van der Waals interactions of ligand and receptor and the internal force-field energy of the ligand, T*Δ*S_Tor_ represents the free energy changes due to conformational energy loss upon ligand binding, *Δ*E_HBond_ is the hydrogen bonding interactions, *Δ*E_HBDesol_ is hydrogen bond donor-acceptor desolvation energy, *Δ*E_SolEl_ represents solvation electrostatic energy upon ligand binding and *Δ*E_HPhob_ is the hydrophobic free energy gain while Q_Size_ is the size correction term proportional to the number of ligand atoms (Neves et al., 2012). The top five predicted ligands with the highest ICM scores retrieved after Stage 2 scanning were selected for further analysis using MD simulations.

### 2.6 Molecular dynamics simulation for the stage 2 receptor-ligand complexes

The topology and coordinate files for the receptor were generated using Amber's Tleap program. The ff14SB force field was used for OR1A2, lipid14 force field was used for the lipid bilayer and the force field parameters for all six ligands (control and 5 predicted) were generated by the general Amber force field (gaff) (Wang et al., 2004) using the Antechamber program. Three minimization steps were performed with the same parameters as mentioned in Section 2.3, to remove steric clashes. The first two steps of minimization were conducted with restraints on the receptor and the ligand while the third step was without any restraint. The minimized complexes were subjected to MD simulations with a protocol similar to that detailed in Section 2.3. Trajectory analysis including root mean square deviation (RMSD), root mean square fluctuation (RMSF) and energy calculations was carried out using the CPPTRAJ (Roe and Cheatham, 2013) module of Amber16, while visualization was done with MDplot (Margreitter and Oostenbrink, 2017).

### 2.7 Molecular mechanics generalized born surface area (MMGBSA) based affinity prediction

The MMGBSA method (Gohlke and Case, 2004) was employed for binding affinity prediction between the predicted ligands and the receptor, OR1A2. The method is based on the force-field utilizing the molecular mechanics, the Generalized Born solvation model and a solvent accessibility method for estimating the binding free energies over the snapshots of MD trajectories. The MMGBSA binding free energy was calculated as in Eq. (5), using the MMPBSA python script in Amber16:

(5) $$\Delta G{{}^{\circ}}_{MMGBSA}={\left(G_{com}\right)}_{i}-{\left(G_{rec}\right)}_{i\;}-\;{\left(G_{lig}\right)}_{i}$$

where ${\left(G_{com}\right)}_{i}$, ${\left(G_{rec}\right)}_{i},\;{\left(G_{lig}\right)}_{i}$ are the average values of $\Delta G{{}^{\circ}}_{MMGBSA}$ for the receptor-ligand complex, the receptor and the predicted ligand for *i* snapshots of the MD trajectories. 500 snapshots were extracted from last 5ns of the MD trajectories. All the MMGBSA calculations were single-trajectory MD simulation i.e. no MD simulations were executed for free ligands.

## 3 Results and discussion

### 3.1 Bovine rhodopsin is the appropriate template for homology modelling of OR1A2

The common methods for template selection are based on local or global sequence similarity (Castleman et al., 2019). However, there exist low sequence identity between available GPCR structures and ORs (Nagarathnam et al., 2014). Thus, sequence identity is not the only measure considered for GPCRs homology modelling as shown in multiple GPCR modelling studies. Phylogenetically close relatives may not always be the best templates and low homology templates have also performed equally well in virtual screening runs (Rataj et al., 2014; Urmi et al., 2017; Perry et al., 2015).

We applied our proposed biophysical approach for GPCR template selection to select the appropriate template for OR1A2. We considered all available GPCR structures listed in the Protein DataBank (PDB). As per GPCRdb (Pándy-Szekeres et al., 2018) statistics, at present, 346 experimental GPCR structures are available in the PDB, representing 64 unique receptors. From these structures, we selected 11 inactive GPCR X-ray structures as candidate templates (Supporting information: Table S2).

A few GPCRs have experimentally resolved structures available in both active and inactive states (Weis and Kobilka, 2018). Agonist binding favours active conformational state, which is less stable than the inactive state. The salt bridge present between the positively charged Arg (R^3.50^) and the negatively charged Aspartic or Glutamic acid (D/E^6.30^) stabilizes the inactive conformation in GPCRs. The binding of the G protein to its binding site, to the intracellular regions of GPCRs, improves the agonist binding affinity. Similarly, agonist binding enhances G protein affinity to the receptor. However, as stated by Weis and Kobilka (Weis and Kobilka, 2018), the “differences between the inactive and active structures in the orthosteric site are remarkably subtle,” so the ligand-binding orthosteric site is preserved once the ligand binds to the inactive GPCR. The ligand-binding site is linked to G protein coupling site through residues within an activation pathway (Zhou et al., 2019). We considered the inactive conformation because biochemically, OR signalling necessitates ligand binding to the inactive receptor (Buck and Axel, 1991) for activation and OR models based on inactive templates have resulted in the discovery of novel ligands for mammalian ORs (Bavan et al., 2014; Abaffy et al., 2018; Baud et al., 2011), and are thus adequate for virtual screening. The selection of candidate templates was based on the best resolved structure (≤2.5 Å) from each of the available GPCRs. Using high resolution structures as templates for homology modelling provides improved side chain and rotamer positions in the model (Crasto, 2010).

We then generated the hydrophobicity profiles for TM regions of each of 11 candidate template and target sequences (OR1A2). Since low sequence identity exists between available GPCR templates and ORs, a hydrophobic similarity measure might be useful for selecting the template for GPCRs. It has been shown previously that the sequences with similar pattern of hydrophobic residues are often structural homologues even if the sequence identity is as low as 7% (Baud et al., 2011). Also, the seven TM domains are the most structurally conserved feature among GPCRs. The hydrophobicity profile is based on hydrophobicity correspondence (HC) which is represented as SSD per residue for each TM. According to the Eisenberg scale, SSD >0.1 per residue indicates poor correspondence between the target and the template. Each of the 11 candidate templates were scored on the basis of SSD per residue for each helix and the overall sequence identity (Supporting information: Table S3). Template 1U19 has minimum sequence identity but good HC with OR1A2 except for TM6. All the templates showing minimum HC among all candidate templates with any TM of OR1A2 were taken to the next step (PDBIDs 5IU4, 6HLP, 1U19, 5ZKC, 2RH1 and 3ODU).

We then calculated query coverage between the six shortlisted templates and OR1A2. Query coverage is an important factor to be considered for homology modelling (Jabeen et al., 2019b). Based on query coverage assessment (Supporting information: Table S4), we shortlisted four templates: bovine rhodopsin (PDBID:1U19) (Crasto, 2010), beta-2 adrenergic receptor (PDBID:2RH1) (Wacker et al., 2017), adenosine receptor A2a (PDBID: 5IU4) (Wolf and Grünewald, 2015) and muscarinic acetylcholine receptor M2 (PDBID: 5ZKC) (Vaidehi and Bhattacharya, 2016). The final alignment between OR1A2 and the candidate template sequences is shown in Supporting information: Fig. S1.

**Fig. 2:**
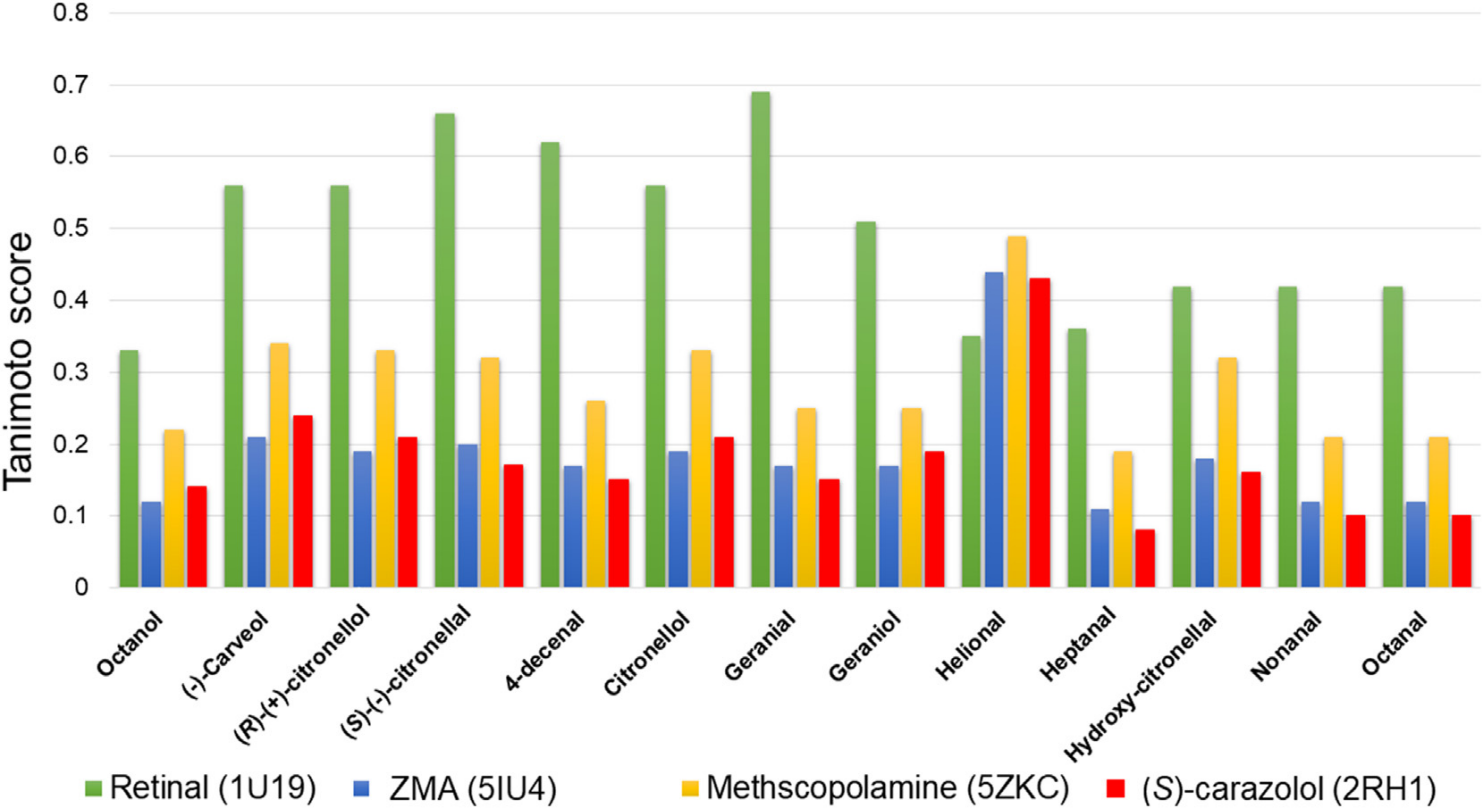
Comparison of ligand profiles for OR1A2 and candidate templates.

Since ligand similarity should also be considered for building good homology models (Nagarathnam et al., 2014), we calculated the similarity between the ligands in the candidate templates and the known ligands of OR1A2, by ranking their Tanimoto scores, based on PubChem fingerprints (shown in (Fig. 2). Retinal (ligand for 1U19; PubchemID: 638015) showed good similarity, with reasonably good Tanimoto scores to known OR1A2 ligands, as compared to ZMA (ligand for 5IU4; PubChemID: 176407), methscopolamine, ligand for 5ZKC; PubChemID: 71183) and (S)-carazolol (ligand for 2RH1: PubChem ID: 13023332).https://pubchem.ncbi.nlm.nih.gov/compound/176407), methscopolamine, ligand for 5ZKC; PubChemID: 71183) and (S)-carazolol (ligand for 2RH1: PubChem ID: 13023332). (Fig. 2).

The traditional “orthosteric” binding site is a site where agonists, a partial agonists and antagonists bind to GPCRs. Agonist binding to the orthosteric site leads to the receptor's activation and allosteric modulation (Wacker et al., 2017). The GPCR orthosteric site lies on its extracellular side and spans mainly TM2-TM7, and the extracellular loop 2 (ECL2) (Wolf and Grünewald, 2015). The activation of a GPCR by an agonist brings about conformational changes within the intracellular side, specifically to TM5, TM6, and TM7, with respect to TM3 (Vaidehi and Bhattacharya, 2016), although the orthosteric binding site undergoes only a slight conformational change (Weis and Kobilka, 2018). The presence of a disulphide bridge between TM3 and ECL2 is highly conserved characteristic feature among class A GPCRs, contributing to receptor stability, and this disulphide bridge is also present within ORs. This feature restricts the conformational change within the extracellular part during GPCR activation, so that the extracellular part stays similar in inactive and active conformations (Venkatakrishnan et al., 2013). The traditional orthosteric binding site within GPCRs comprises 24 residue positions, namely 3.28, 3.29, 3.32, 3.33, 3.36, 3.37, 4.52, 5.39. 5.40, 5.43, 5.44, 5.47, 5.53, 6.44, 6.48, 6.51, 6.52, 6.55, 6.58, 7.31, 7.34, 7.38, 7.41, and 7.42 (Chan et al., 2019), in the Ballesteros–Weinstein numbering scheme for GPCRs (Ballesteros and Weinstein, 1995). According to the site directed mutagenesis data available for human ORs (Launay et al., 2012; Keller et al., 2007; Noe et al., 2017; Gelis et al., 2012; Wolf et al., 2017; Geithe et al., 2017; Schmiedeberg et al., 2007; Ahmed et al., 2018) and mouse ORs (Yu et al., 2015; Baud et al., 2015; Thach et al., 2017) residue positions 3.33, 3.36, 3.37, 5.43, 5.47, 6.48, 6.51, 6.52, 6.55, 7.41, and 7.42 are important for ORs as well. We computed the similarity between GPCR orthosteric binding site of all templates and OR1A2 using GPCRtm (Rios et al., 2015), which is an amino acid substitution matrix specifically designed for GPCRs. The template 1U19 showed the maximum score (2) followed by 5ZKC (-5), 5IU4 (−7), and 2RH1 (−16).

We then compared the ligand-binding site residues of the templates with the residues important for ligand binding (hot spots) in OR1A2 in detail. OR1A2 has mutagenesis data available for only five residue positions (A18^3.36^, K109^3.37^, S112^3.40^, S155^4.56^ and V205^5.46^) which is insufficient for binding site analysis. Thus, we considered the mutagenesis data for OR1A1, a closest homolog of OR1A2 (Sequence identity: 83.5%) as well, for the binding site analysis. There is 46% similarity between OR1A2 and 1U19 in the hot spot residues, followed by 2RH1 (38%), 5ZKC (23%) and 5IU4 (15%). Moreover, positions 3.36, 3.37, 5.46, and 6.48 are important for ligand binding in 1U19 and in 5ZKC. Binding pockets of all four templates are dissimilar but positions 3.33 and 6.48 are important for ligand binding in all four templates (Supporting information: Table S5).

In the final stage of template selection, we generated 250 models for OR1A2. 50 models were generated for each of the four selected templates and 50 models were generated using multiple templates (as per alignment shown in Supporting information: Fig. S1). We performed ligand-supported modelling for each template. Final models were selected after examining the DOPE score, visual inspection for structural features like disulphide bonding, integrity of the seven TMs and Ramachandran plot analysis. After modelling, the included ligand was removed from each of the templates and (S)- (−)-citronellal was docked into the predicted binding of each model. The OR1A2 site-directed mutagenesis data for (S)- (−)-citronellal was used as a control, to check the presence of important ligand-binding residues within the predicted binding pockets of the models during docking runs. We further refined the binding pocket by docking two more experimentally known ligands for OR1A2 that are helional and octanal. We were able to recover 7/12 hot spot residues using 1U19-based OR1A2 model followed by 6/12 with 5ZKC model, 5/12 with 2RH1, 3/12 for multiple template-based model and none for 5IU4 based model (Supporting information: Fig. S2). The in-depth comparison of resolution, HC between TMs of target and candidate templates, ligand profiles, and binding pocket analysis supported a 1U19-based model for further analysis (Supporting information: Table S6). Previously, the inactive conformation of bovine rhodopsin has been used for OR model building on the basis of which mutagenesis data has been derived for ORs such as OR1A1, OR1A2 (template: 1F88) (Schmiedeberg et al., 2007), OR2AG1 (template: 1U19) (Venkatakrishnan et al., 2013), and OR51E2 (template: 1U19) (Wolf et al., 2017), consistent with the biochemistry of OR signalling (Buck and Axel, 1991). Our template selection for OR1A2 through the biophysical approach described here is in accordance with earlier studies selecting bovine rhodopsin as the appropriate template for homology modelling of ORs.

### 3.2 Homology model refinement for structure based virtual screening

**Fig. 3:**
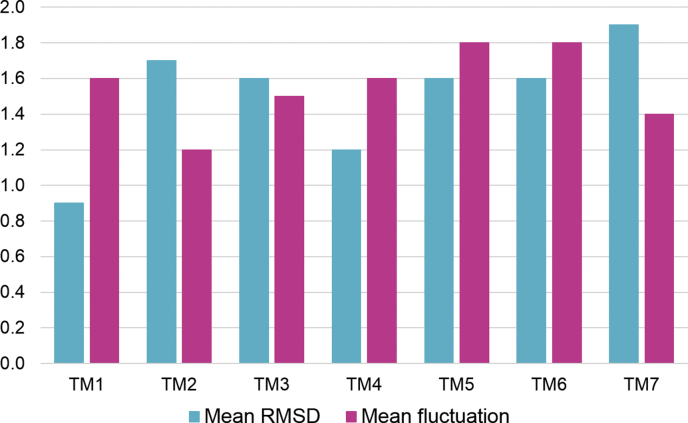
Mean backbone atom deviation and mean atomic fluctuation for residues within 7 TM helices for 1U19-based *apo* model, in Å.

Though GPCRs crystal structures show better performance in docking than homology models, refined homology models combined with induced fit docking have shown comparable performance to crystal structures (Chan et al., 2019). Therefore, we performed the overall refinement of 1U19-based model through MD simulations. We refined models both in *apo* and *holo* forms. Initially, 89.9% of the residues were in favoured regions according to the Ramachandran plot embedded into PSVS server (Ballesteros and Weinstein, 1995), which were latterly improved to 91.2% with the refinement. The overall RMSD values for the backbone atoms of both 1U19-based *apo* model and 1U19-based *holo* model indicates that the receptor underwent rapid deviations during the first 4 ns (Supporting information: Fig. S3). The standard deviation of backbone RMSD for the last 10ns of MD production phase was 0.15 for 1U19-based *apo* model and 0.11 for 1U19-based *holo* model showing the stability of both models. For both models, greater atomic fluctuations were observed in ICL2 and ECL2 (Supporting information: Fig. S4), compared to the other structural elements. Also, the residues within the individual TM regions did not show much deviations from the initial homology model (Supporting information: Fig. S5). The mean deviation for all the TM regions was within 2 Å with the maximum mean RMSD value of 1.9 Å for TM7 in 1U19- based *apo* model. All TM regions are showing stability at the end of MD production (Fig. 3).

We analysed the predicted binding pockets for both models. After *holo* simulation, the ligand-binding pocket was considerably narrow and relocated towards the OR channel entrance, compared to the *apo* conformation (Supporting information: Fig. S6). This finding is consistent with a recent study for two GPCRs, D2 dopamine receptor (D2R) and 5-HT2A serotonin receptor (5-HT2AR) wherein the authors showed binding site divergence from the initial homology model in *holo* simulated models (Launay et al., 2012). We docked known ligands to 1U19-based *holo* simulated model and could not recover most of the interactions with the hot spot residues. We were able to recover only one hotspot residue i.e. Y251^6.48^ after the refinement of the *holo* 1U19-based OR1A2 model (Supporting information: Fig. S7). The *holo* model simulation system showed less flexibility due to the bound ligand, as noted for rat muscarinic M3 receptor (Lupala et al., 2018) and for CCR5 (Salmas et al., 2015). We also performed VS with 1U19-based *holo* simulated model. We took the top two predicted ligands and carried out MD simulation for their complexes with OR1A2. Both ligands were displaced from the predicted binding pocket and lacked most of the hotspot residues at the end of 40ns MD simulation. Therefore, we did not proceed with the *holo* simulated model (Supporting information: Fig. S8) and selected 1U19-based *apo* refined model for SBVS.

### 3.3 Two-stage screening and docked metabolites

The two computational approaches used in computer aided drug discovery include SBVS and ligand based virtual screening (LBVS). The choice of method is largely determined by availability of the information. Combining the methods for the two approaches are shown to outperform the individually applied VS method (Svensson et al., 2012). One of the common methods for LBVS is the generation of 3D pharmacophore. The generation of good ligand-based 3D pharmacophore depends on molecular alignment which is a challenging task. Since the similar chemical structures often bind to the similar proteins (Grigoryan et al., 2010), retrieving the molecules that structurally align to the known agonists would assist in discovery of novel agonists for a receptor. We have used the APF superposition method which is based on Monte Carlo minimization of atomic property field potentials to generate the 3D ligand pharmacophore. The APF method has shown competitive performance on benchmarking datasets (Totrov, 2008) and shows better performance than traditional pocket based docking methods for GPCRs (Kufareva et al., 2012).

The ligand pharmacophore was built by super positioning the experimentally known ligands for OR1A2 and predominantly featured two properties that are lipophilic and charge property (Supporting information: Fig. S9). Each property was selected for pharmacophore building when exhibited by 75% of the ligands. Virtual screening using APF based pharmacophore retrieved 438 metabolites (Supporting information: Table S7) based on lipophilic and charge properties. These metabolites were structurally similar to experimentally known agonists for OR1A2. In order to further screen these compounds SBVS was used. Since the ICM docking score of the control, (*S*)-(−)-citronellal and OR1A2 is −13.686, we used a threshold value of ICM docking score of at least −10, for virtual screening. The predicted binding pocket for OR1A2 comprises of a combination of hydrophobic, polar and charged residues depicting the ability to accommodate variety of ligands (Supporting information: Fig. S6). The predicted binding pocket is comprised of residues from TM2, TM3, TM5, TM6 and TM7. The canonical binding pocket for class A GPCRs is proposed to be formed by residues lying in TM3, TM5, TM6 and TM7 (de March et al., 2015b).

The ICM docking scores of the 13 known OR1A2 ligands (Supporting information: Table S8) were used to estimate the ICM docking score threshold for SBVS. Compounds that were docked within the vicinity of binding pocket and had docking score less than the established threshold were selected for further analysis. The top five compounds (Supporting information: Table S9 and Fig. S10); labelled **1**–**5**, with the control molecule, (*S*)-(−)-citronellal labelled **6**) comprising 14-methylhexadecanoic acid, pentadecanoic acid, hexadecanedioic acid, 2-hydroxytetradecanoic acid and palmitic acid having ICM score < −10 were selected for further analysis using MD simulations. Pentadecanoic acid is the biological marker for dairy food intake and have association with lowering the type 2 diabetes risk (Imamura et al., 2018). Further, it has been suggested as a biomarker for nonalcoholic steatohepatitis (NASH) in non-alcoholic fatty liver disease (NAFLD) patients (Yoo et al., 2017). Hexadecanedioic acid has known to be activated in human liver (Pettersen and Aas, 1974) and act as a substrate for OATP1B1, a liver transporter for metabolites (Yee et al., 2016). Moreover, it is also known to be associated with high blood pressure (Menni et al., 2017). Palmitic acid is used in the industry for soap production, cosmetics and other uses. It may also be involved in lipid deposition in HepG2 cells (Zhou et al., 2018) and have possible implications in obesity, type 2 diabetes, cardiovascular diseases and cancer (Mancini et al., 2015).

### 3.4 Dynamics and simulation analysis of the complexes

Docking identified potential receptor binding compounds and clues for their binding modes to the receptor. To assess the stability of these complexes, MD was executed. The receptor-ligand complexes were subjected to MD simulation with the same protocol as followed for refinement of the receptor. The trajectories were analysed using CPPTRAJ. RMSD plots (Supporting information: Fig. S11) for complexes of OR1A2 with (*S*)- (−)-citronellal and hexadecanedioic acid indicates the stability of these complexes throughout the simulation time with RMSD value below 2 Å. The OR1A2:14-methylhexadecanoic acid complex showed deviations during the first 15 ns, but eventually stabilized, with a mean RMSD value of 2 Å. The OR1A2:2-hydroxytetradecanoic acid complex showed the maximum deviations from the initial structure, with a maximum value of 3.16 Å and started to stabilize after 30 ns, with a mean RMSD value of 2.3 Å. All the predicted ligands have shown slight conformational changes, from their initial orientations, illustrating their rearrangements within the binding pocket to attain stability (Supporting information: Fig. S12). These rearrangements are also evident from their post-simulation interaction sites differing from their initial docked sites (Supporting information: Fig. S13). The predicted ligands, excepting 2-hydroxytetradecanoic acid, tend to adopt more stable conformations and locations, by forming hydrogen bonds with the residues within the receptor's binding pocket. We also analysed the deviations of control and predicted ligands within the receptor binding pocket. (*S*)-(−)-citronellal (control) showed the mean deviation of 0.6 Å while 2-hydroxytetradecanoic acid and pentadecanoic acid showed the maximum deviation, with a mean value of 2.3 Å. Hexadecanedioic acid, 14-methylhexadecanoic acid and palmitic acid had mean deviations of 2.0 Å, 2.1 Å and 2.1 Å respectively. 2-hydroxytetradecanoic acid interacts primarily with ECL2 (Supporting information: Fig. S11). The RMSF analysis (Supporting information: Fig. S14) indicated the high fluctuations within residues at the N-terminus of the receptor. The residues within ICL2 and ECL2 also showed fluctuations, as seen in the 3D view (Supporting information: Fig. S13) but the mean fluctuations were decreased as compared to the unbound homology model. Thus, ligand binding has enabled the receptor to become more stable over the production phase of MD simulations. The RMSD values of all the complexes indicate no drastic changes from the initial conformation after the 40ns MD simulation and all the complexes have attained stability by the end of 40ns MD production.

**Fig. 4:**
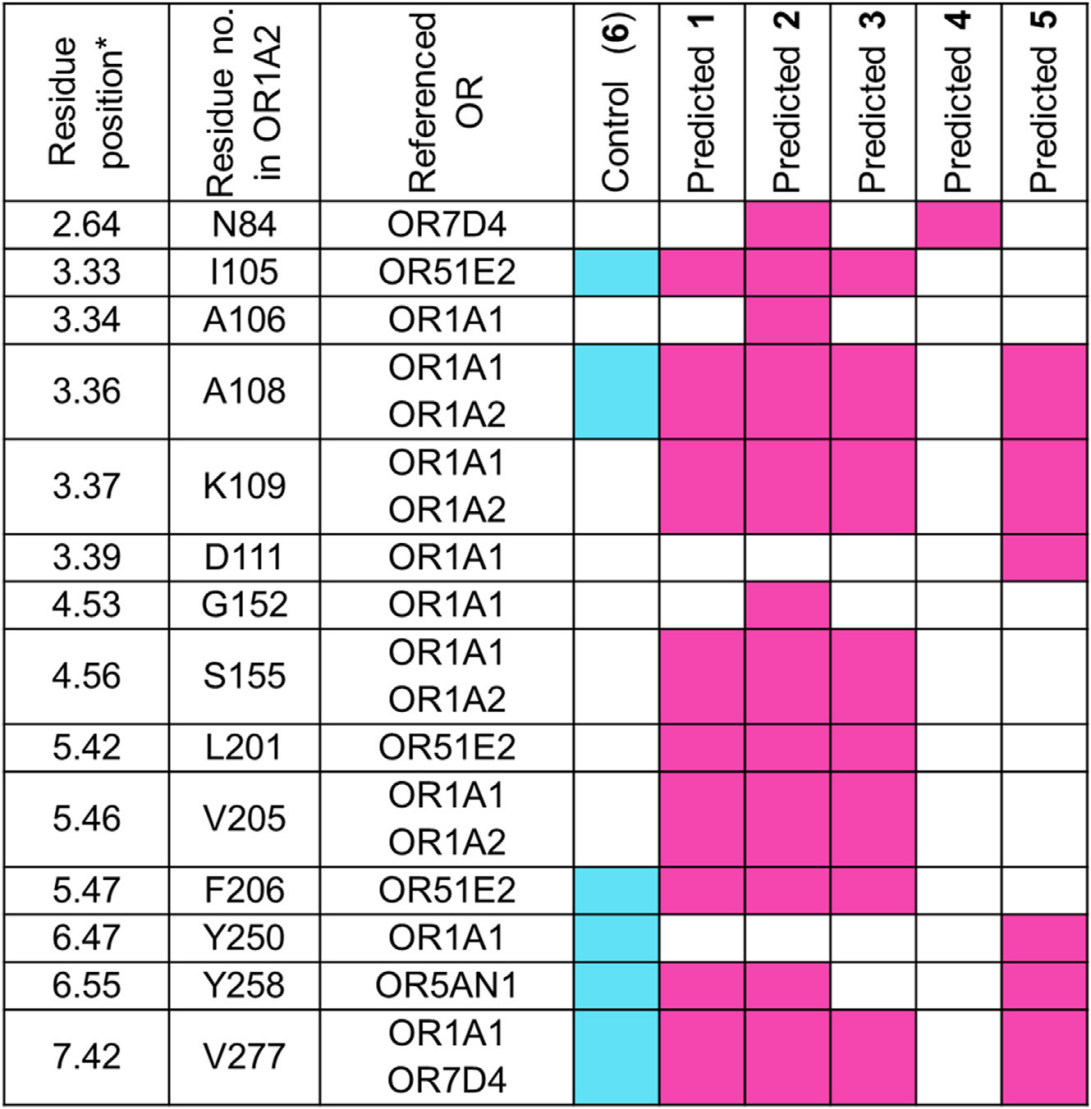
OR1A2 ligand binding positions having correspondence with mutagenesis data for various ORs; pink shading represents overlapping positions in predicted agonists while blue shading represents overlapping positions in control (**6**: (S)-(−)-citronellal). Predicted **1** is pentadecanoic acid, predicted **2** is hexadecanedioic acid, predicted **3** is 14-methylhexadecanoic acid, predicted **4** is 2-hydroxytetradecanoic acid while predicted **5** is palmitic acid. Residue positions are numbered according to the Ballesteros-Weinstein residue numbering scheme (Ballesteros and Weinstein, 1995).

We further analysed the residues within OR1A2 that are interacting with predicted ligands and their correspondence with the positions having mutagenesis data available for other ORs. Currently, site directed mutagenesis data is available for OR1A1, OR1A2, OR2M3, OR2T11, OR5AN1, OR7D4, and OR51E2 (Jabeen et al., 2019a). According to the available mutagenesis data for OR1A2, positions 3.36 (A108), 3.37 (K109), 4.56 (S155) and 5.46 (V205) have crucial roles within the ligand-binding pocket and 4 of the top 5 predicted ligands, except 2-hydroxytetradecanoic acid show interactions with these residues as evident from Fig. 4 and Supporting information: Fig. S13.

### 3.5 Binding free energy calculation

Binding free energy calculation based on MD trajectories predicts the binding affinities between the ligands and the receptors. We have performed the binding affinity prediction using MMGBSA. molecular mechanics Poisson–Boltzmann surface area (MMPBSA) and MMGBSA have been successfully applied for improving the virtual screening results but GB solvation models are more accurate than PB (Genheden and Ryde, 2015). 500 snapshots from the last 5ns simulation for each complex were analysed for energy convergence to estimate the energetic stability of these complexes. The binding free energies estimated for each complex is shown in Table 1. Notably, MM van der Waals values are contributing markedly to the binding energy for all complexes. The contribution of the electrostatic energy to the Δ*G*_bind_ is the least for OR1A2:(*S*)-(−)-citronellal, followed by OR1A2:14-methylhexadecanoic acid and OR1A2:pentadecanoic acid complexes. For OR1A2:hexadecanedioic acid complex, contribution of the electrostatic energy to the Δ*G*_bind_ is very favourable (most negative), contributing maximally to Δ*G*_bind_. The binding affinity of the predicted compounds **1**–**5** is better (lower) than that of the control compound ((*S*)-(−)-citronellal, **6**).

**Table 1:** Binding free energy (ΔG_bind_ in kcal mol^−1^) and other energy components for the control and predicted complexes.

OR1A2 Complex with	**ΔE**_**vdW**_	**ΔE**_**Ele**_	**ΔE**_**Sol**_	**ΔG**_**gas**_	**ΔG**_**bind**_
(S)-(−)-citronellal (control, **6**)	−26.28	−0.87	7.3753	−27.15	−19.78
Pentadecanoic acid (**1**)	−40.20	−8.20	12.42	−48.41	−35.98
Hexadecanedioic acid (**2**)	−39.28	−40.27	42.19	−79.55	−37.36
14-methylhexadecanoic acid (**3**)	−45.84	−2.21	8.27	−48.06	−39.78
2-hydroxytetradecanoic acid (**4**)	−33.50	−26.03	23.05	−59.54	−36.48
Palmitic acid (**5**)	−41.57	−18.49	17.86	−60.06	−42.19

ΔE_vdW:_ van der Waals energy contribution from MM; ΔE_Ele:_ Electrostatic energy; ΔE_Sol_: Sum of the polar and non-polar solvation energies of the molecules estimated by GB; ΔG_gas_: Gas phase relative free energy; ΔG_bind_ : Final estimated binding free energy.

## 4 Conclusions

Decoding the complex combinatorial code of ORs is yet challenging provided the huge number of odors and large number of ORs but essential to elucidate the olfactory recognition process. *In silico* approaches are being successfully applied in exploring the chemical space for deciphering the molecular receptive range of ORs. In this study we have screened the library of 5942 compounds against OR1A2 by computational means to identify the putative ligands for the receptor. In our protocol we have combined the LBVS and SBVS. Further we have used a combination of parameters including a novel measure of hydrophobicity similarity matching for template selection. We suggested that Bovine Rhodopsin (PDBID:1U19) is the best template to predict the OR1A2 homology model. The top predicted ligands retrieved after two-stage screening are acidic in nature and are structurally similar to the experimentally known potent ligands for the receptor. Moreover, the predicted ligands are energetically stable within the binding pocket of homology model and have better binding energies than experimentally validated ligands according to the MMGBSA energy calculations. Therefore, we recommend the *in-vitro* testing of the predicted compounds against the OR1A2.

## Author contributions

**Amara Jabeen**, Conceived and designed the analysis, Collected the data, Contributed data or analysis tools, Performed the analysis, Wrote the paper, **Ramya Vijayram**: Contributed data or analysis tools, **Shoba Ranganathan**: Conceived and designed the analysis, Wrote the paper

## Declaration of competing interest

The authors declare that they have no known competing financial interests or personal relationships that could have appeared to influence the work reported in this paper.
