# Supplementary material for: A two-stage computational approach to predict novel ligands for a chemosensory receptor
Source: Curr Res Struct Biol. 2020 Oct 9;2:213–21. doi: 10.1016/j.crstbi.2020.10.001 (PMC8244491; doi:10.1016/j.crstbi.2020.10.001)
Supplement: Multimedia component 1 [file mmc1.pdf]

A two-stage computational approach to predict novel ligands for a chemosensory receptor

Amara Jabeen, Ramya Vijayram, Shoba Ranganathan

Supplementary Information

Contents

Table S1: Experimentally known ligands for OR1A2 2
Table S2: Templates selected in step 1 with resolution ≤ 2.5Å 2
Table S3: Hydrophobicity correspondence and sequence identity between 11 templates 3
Table S4: Query coverage of alignment between OR1A2 sequence and templates 3
Figure S1: Multiple sequence alignment between OR1A2 and selected templates 4
Table S5: Comparison between OR1A2 hot spot residues and corresponding residues in candidate templates 5
Figure S2: Receptor-ligand interactions after local refinement 6
Table S6: Parameters for template selection: 7
Figure S3: RMS deviation (for Cα atoms) for OR1A2 8
Figure S4: Positional atomic fluctuation for the backbone atoms of OR1A2 receptor 8
Figure S5: Backbone atoms RMSDs of 7 TM regions for OR1A2. 9
Figure S6: The predicted binding pocket 10
Figure S7: 1U19-based-*holo*-OR1A2 model interactions with (S)-(-)-citronellal 10
Figure S8: Interacting residues of the complexes with 1U19-based-*holo* model. 11
Figure S9: APF superposition based pharmacophore 12
Table S7: Compounds retrieved after stage 1 screening 13
Table S8: Docking scores for known ligands of OR1A2 17
TableS9: Metabolites retrieved after Stage-2 scanning with APF and docking scores 18
Figure S10: Chemical structures of the top five metabolites (1-5) 19
Figure S12: RMSD of the control ((*S*)- (-)-citronellal) and predicted ligands for OR1A2 ... 20
Figure S13: Interacting residues of the complexes before and after molecular dynamics.. 22
Figure S14: RMSF of the control ((*S*)- (-)-citronellal) and predicted ligands for OR1A2 23

Table S1: Experimentally known ligands for OR1A2

Pubchem_CID	Odorant name	Chemical nature	EC_{50} (μM) [1]
957	Octanol	Alcohol	60.4 ± 15.0
7438	(-)-Carveol	Terpene	16.7 ± 5.2
101977	(*R*)-(+)-citronellol	Terpene	81.1 ± 6.2
443157	(*S*)-(-)-citronellal	Terpene	2.4 ± 0.7
61875	4-decenal	Aldehyde	16.5 ± 3.8
8842	Citronellol [2]	Terpene	Not reported
638011	Geranial	Terpene	14.7 ± 1.9
637566	Geraniol	Alcohol	>0.1
64805	Helional	Aldehyde	3.4 ± 1.1
8130	Heptanal	Aldehyde	1.7 ± 0.9
7888	Hydroxy-citronellal	Terpene	3.9 ± 0.4
31289	Nonanal	Aldehyde	127.4 ± 30.7
454	Octanal	Aldehyde	4.3 ± 1.3

Table S2: Templates selected in step 1 with resolution ≤ 2.5Å

Receptor	PDBID	Resolution (Å)
AA2AR_HUMAN	5IU4	1.7
OPRD_HUMAN	4N6H	1.8
OX2R_HUMAN	5WQC	1.9
C5AR1_HUMAN	6C1R	2.2
CCR5_HUMAN	5UIW	2.2
NK1R_HUMAN	6HLP	2.2
OPSD_BOVIN	1U19	2.2
ACM2_HUMAN	5ZKC	2.3
ADRB2_HUMAN	2RH1	2.4
CXCR4_HUMAN	3ODU	2.5
TA2R_HUMAN	6IIU	2.5

Table S3: Hydrophobicity correspondence (HC) and sequence identity between 11 candidate templates selected in stage 1 and sequence identity: minimum HC values are in red font.

PDBID	Sequence identity (%)	Hydrophobicity correspondence (HC)						
		TM1	TM2	TM3	TM4	TM5	TM6	TM7
5IU4	22.7	0.131	0.075	0.062	0.076	0.074	0.096	0.099
4N6H	24.1	0.114	0.027	0.039	0.105	0.052	0.312	0.086
5WQC	20.5	0.062	0.025	0.046	0.251	0.059	0.233	0.052
6C1R	26.0	0.055	0.042	0.057	0.084	0.166	0.155	0.08
5UIW	22.0	0.052	0.039	0.186	0.09	0.066	0.3	0.12
6HLP	22.9	0.079	0.032	0.062	0.101	0.02	0.153	0.039
1U19	18.8	0.095	0.023	0.079	0.045	0.032	0.178	0.091
5ZKC	20.8	0.103	0.029	0.029	0.143	0.159	0.205	0.071
2RH1	25.0	0.063	0.054	0.031	0.075	0.149	0.104	0.022
3ODU	26.0	0.035	0.244	0.069	0.123	0.037	0.221	0.058
6IIU	25.5	0.041	0.033	0.147	0.138	0.021	0.117	0.084

Table S4: Query coverage of alignment between OR1A2 sequence and templates selected in step 2: templates selected in step 3 are in red font color.

Receptor	PDBID	Query coverage
AA2AR_HUMAN	5IU4	91
NK1R_HUMAN	6HLP	20
OPSD_BOVIN	1U19	90
ACM2_HUMAN	5ZKC	71
ADRB2_HUMAN	2RH1	71
CXCR4_HUMAN	3ODU	46

```
                                                           TM1       1.50
OR1A2   MKKENQSFNLDFILLG-------------VTSQQ---EQNNVFFVIFLCIYPITLTGNLL  45
1U19    MNGTEGPNFYV-PFSNKTGVVRSPFEA-PQYYLAEPWQFSMLAAYMFLLIMLGFPINFL  58
5IU4    MPI----------------------------------MGSSVYITVELAIAVLAILGNVL  27
5ZKC    MNNSTNSSNNS-LAL--------------TSPY--KTFEVVFIVLVAGSLSLVTIIGNIL  44
2RH1    MGQPGNGSAFL-LAPNGS---HAPDHDVTQER--DEVWVVGMGIVMSLIVLAIVFGNVL  54

          TM1                      2.50       TM2                 TM3
OR1A2   ILAICADIRLHNPMYFLLANLSLVDIIFSSVTIPKVLANHLLGSKFISFGGCLMQMYFM  105
1U19    LYVTVQHKKLRTPLNYILLNLAVADLFMVFGGFTTTLYTSLHGYFVFGPTGCNLEGFFA  118
5IU4    CWAVWLNSNLQNVTNYFVVSLAAADIAVGVLAIPFAITIST--GFCAACHGCLFIACFV  85
5ZKC    MVSIKVNRHLQTVNNYFLFSLACADLIIGVFSMNLYTLYTVIGYWPLGPVVCDLWLALD  104
2RH1    ITAIAKFERLQTVTNYFITSLACADLVMGLAVVPFGAAHILMKMWTFGNFWCEFWTSID  114

           TM3        3.50                         4.50     TM4
OR1A2   ALAKADSYTLAAMAYDRAVAISCPLHYTTIMSPRSCILLIAGSWVIGNTSALPHTLLTA  165
1U19    LGGEIALWSLVVLAIERYVVVCKPMSNF-RFGENHAIMGVAFTWVMALACAAPPLVGWS  177
5IU4    VLTQSSIFSLLAIAIDRYIAIRIPLRYNGLVTGTRAKGIIAICWVLSFAIGLTPMLGWN  145
5ZKC    VVSNASVMNLLIISFDRYFCVTKPLTYPVKRTTKMAGMMIAAAWVLSFILWAPAILFWQ  164
2RH1    LCVTASIETLCVIAVDRYFAITSPFKYQSLLTKNKARVIILMVWIVSGLTSFLPIQMHW  174

                                                       TM5      5.50
OR1A2   LSFCGNQEVANFY-------CDIMPLLKLSCSDV---HFNVKMMYLGVGVFSLPLLCII  215
1U19    YIPEGM----QCS--------CGID---YYTPHEET--NNESFVIYMFVVHFIIPLIVIF  221
5IU4    CGQPKEGKNHSQGCGEGQVACLFEDVV-----------PMNYMVYFNFFACVLVPLLLML  195
5ZKC    IVGVRTVEDG---------ECYIQFF------------SNAAVTFGTAIAAFYLPVIIMT  204
2RH1    RATHQEA----INCYANETCCDFF- ------------TNQAYAIASSIVSFYVPLVIMV  217

           TM5
OR1A2   SYVQVFSTVFQVPST---------------------------------------------  230
1U19    CYGQLVFTVKEAAAQQQES-----------------------------------------  240
5IU4    VYLRIFLAARRQLKQMESQPLPGERARSTL------------------------------  225
5ZKC    LYWHISRASKSRIKKDKKEPVANQDPVSPSLVQGRIVKPNNNNMPSSDDGLEHNKIQNG  264
2RH1    VYSRVFQEAKRQLQKIDKSEGRFHV-----------------------------------  242

                                                                 TM6
OR1A2   --------------------------------------------------------KSLF  235
1U19    ----------------------------------------------------ATTQKAEK  249
5IU4    --------------------------------------------------------QKEV  230
5ZKC    IRIGTKTPKSDSCTPTNTTVEVVGSSGQNGDEKQNIVARKIVKMTKQPAKKKPPPSREK  384
2RH1    ---------------------------------QNLSQVEQDGRTGHGLRRSSKFCLKEH  270

              TM6                                    TM7
                         6.50                                  7.50
OR1A2   AFCTCGSHLTVVFLYYGTTMGMYFRPLTS-----YSPKDAVITVMYVAVTPALNPFIYS  290
1U19    VTRMVIIMVIAFLICWLPYAGVAFYIFTHQGS-DFGPIFMTIPAFFAKTSAVYNPVIYI  308
5IU4    AAKSLAIIVGLFALCWLPLHIINCFTFFCPDCSHAPLWLMYLAIVLSHTNSVVNPFIYA  290
5ZKC    VTRTILAILLAFIITWAPYNVMVLINTFCAPC--IPNTVWTIGYWLCYINSTINPACYA  442
2RH1    ALKTLGIIMGTFTLCWLPFFIVNIVHVIQDNL--IRKEVYILLNWIGYVNSGFNPLIYC  328

OR1A2   RNWDMKAALQKLFSKRISS| 309
1U19    MNKQFRNCMVTTLCCGK    348
5IU4    RIREFRQTFRKIIRSHVLR  360
5ZKC    CNATFKKTFKHLLMCHY    459
2RH1    SPDFRIAFQELLCLRR     344
```

Figure S1: Multiple sequence alignment between OR1A2 and selected templates: ligand binding residues for templates and residues with mutagenesis data for OR1A1 are in green color, residues with mutagenesis data for OR1A2 are in purple color. Centre residues are in red color. cysteine residues forming disulphide bridge are in blue color. Transmembrane helices are highlighted as gray. Residues 264 to 324 in 5ZKC are not shown in the above alignment as they were not aligning to any template and target.

Table S5: Comparison between OR1A2 hot spot residues and corresponding residues in candidate templates: similar residues are in red font color, Ballesteros Weinstein (B.W.) positions important for ligand binding in OR1A1 are in italics

B.W. position	OR1A2	5IU4	1U19	5ZKC	2RH1
3.34	A	V	L	V	L
3.36	A	T	G	S	V
3.37	K	Q	E	N	T
3.39	D	S	A	S	S
3.40	S	I	L	V	I
4.53	G	S	A	S	S
4.56	S	I	C	L	T
5.46	V	C	H	A	S
6.47	Y	C	C	T	C
6.48	Y	W	W	W	W
6.55	Y	N	A	V	N
7.41	Y	L	F	L	I
7.42	V	S	A	C	G

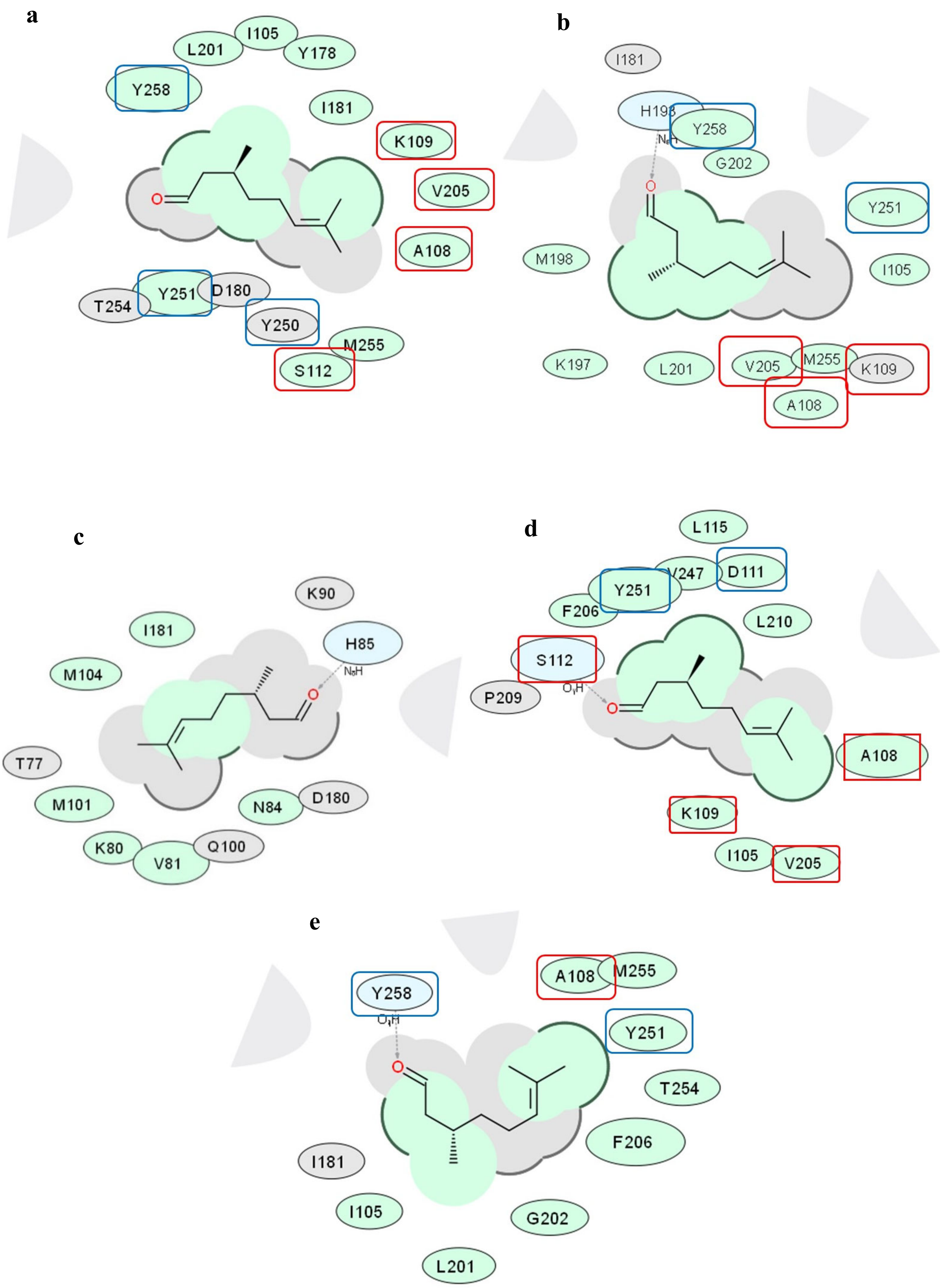

Figure S2: Receptor-ligand interactions after local refinement for (a) 1U19-based model, (b) 2RH1-based model, (c) 5IU4-based model, (d) 5ZKC-based model and (e) multiple template-based model. The important ligand binding residues having mutagenesis data for OR1A2 are boxed in red while for OR1A1 are boxed in blue.

Table S6: **Parameters for template selection:** cross indicating best ranked for each parameter

Template	Res.	SI	QC	LP	HC-TM1	HC-TM2	HC-TM3	HC-TM4	HC-TM5	HC-TM6	HC-TM7	LBS	Σ
5IU4	X		X							X			3
4N6H													0
5WQC													0
6C1R		X											1
5UIW													0
6HLP									X				1
1U19				X		X		X				X	4
5ZKC							X						1
2RH1												X	1
3ODU		X			X								2
6IIU													0

Res: Resolution, SI & QC: Sequence identity and query coverage, LP: Ligand profile similarity, LBS: Ligand binding site.
HC: hydrophobicity correspondence, TM: transmembrane helix
Σ: overall score for all parameters

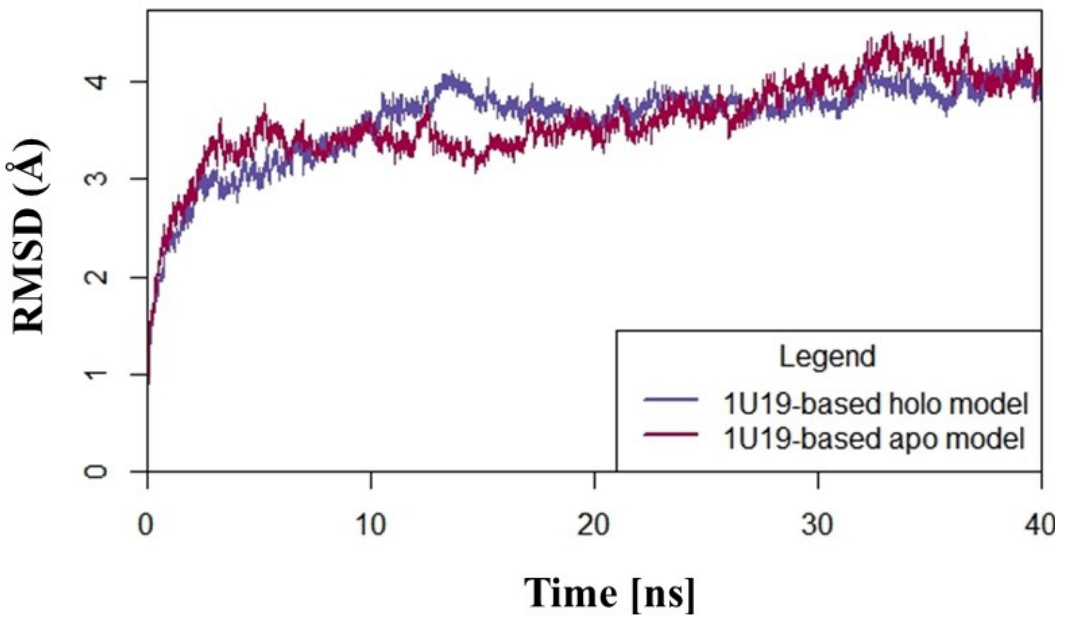

Figure S3: **RMS deviation (for Cα atoms) for OR1A2**

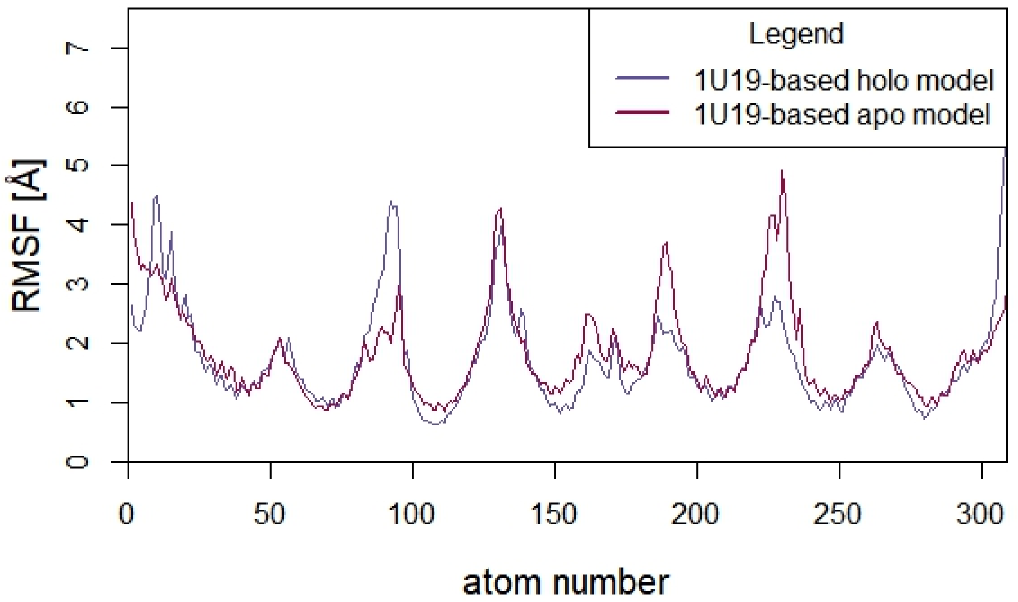

Figure S4: Positional atomic fluctuation for the backbone atoms of OR1A2 receptor

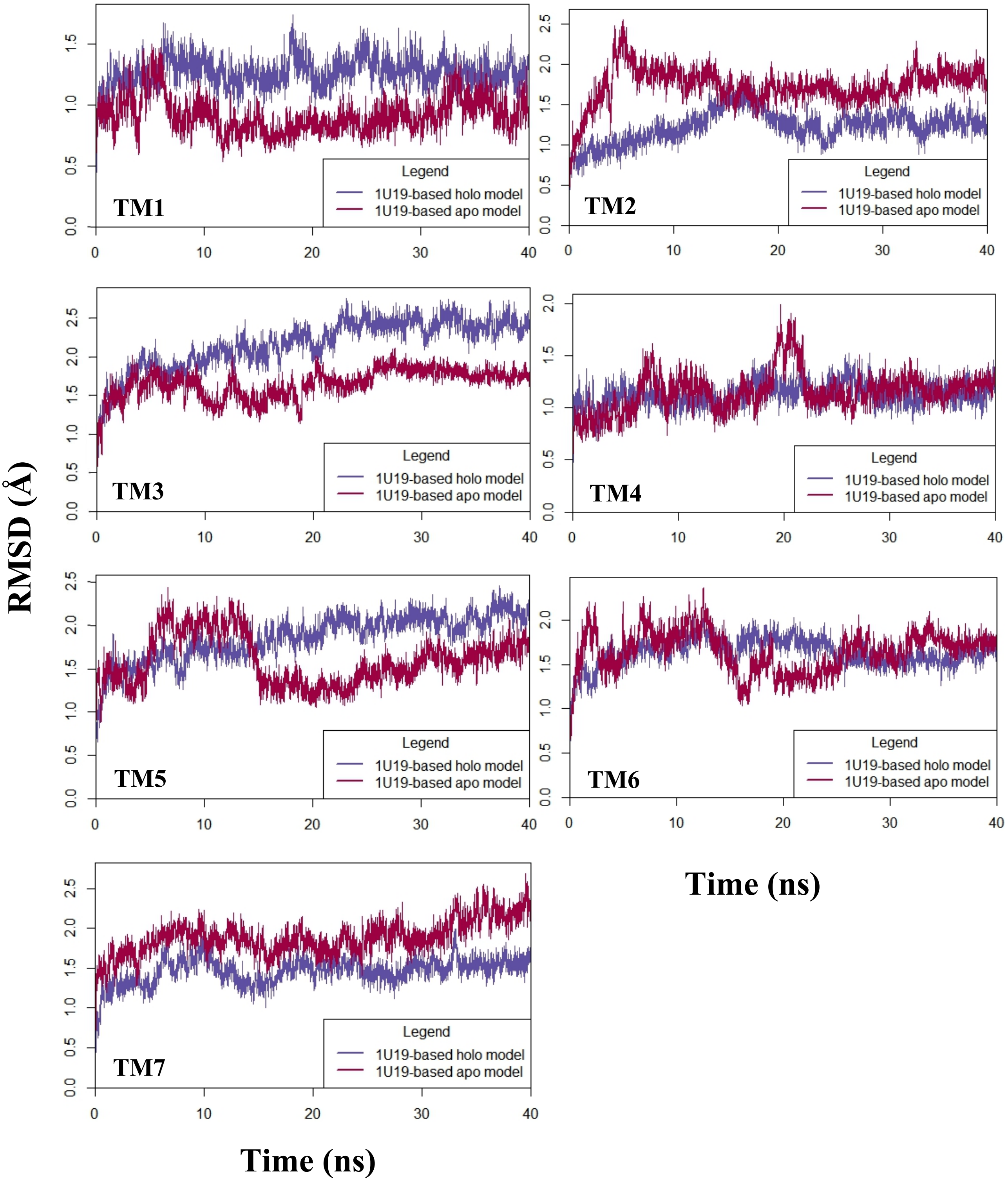

Figure S5: Backbone atoms RMSDs of 7 TM regions for OR1A2 showing the comparison of initial conformation over molecular dynamics simulation time.

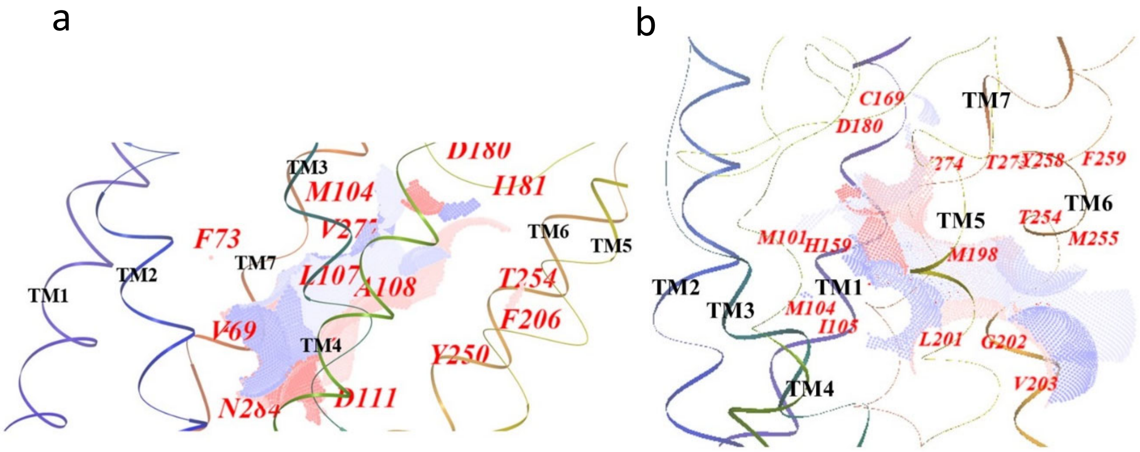

Figure S6: **The predicted binding pocket** for (a) 1U19-based *apo* model for OR1A2 and (b) 1U19-based *holo* model for OR1A2: The binding pocket is colored on the basis of hydrophobicity (Blue representing hydrophillic residues while red representing hydrophobic residues). The receptor is colored from N-terminus (Blue) to C-terminus (orange). The predicted binding site contains residues from TM2, TM3, TM5, TM6 and TM7

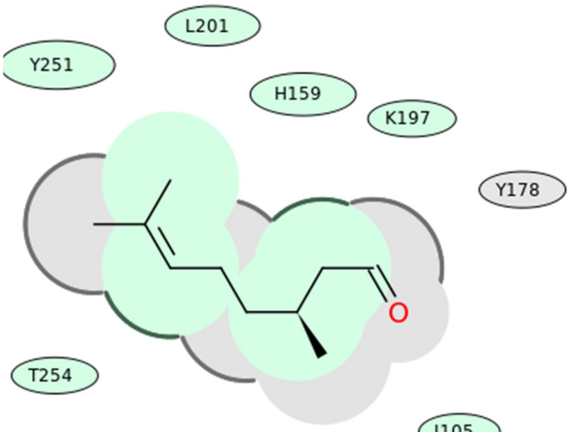

Figure S7: 1U19-based-*holo*-OR1A2 model interactions with (S)-(-)-citronellal recovered only one hotspot residue i.e. Y251.

Interacting residues and type of interaction before MD production	Interacting residues and type of interaction after MD production (40ns)

Lysophosphatidylethanolamine
(PubChem ID: 52925131)

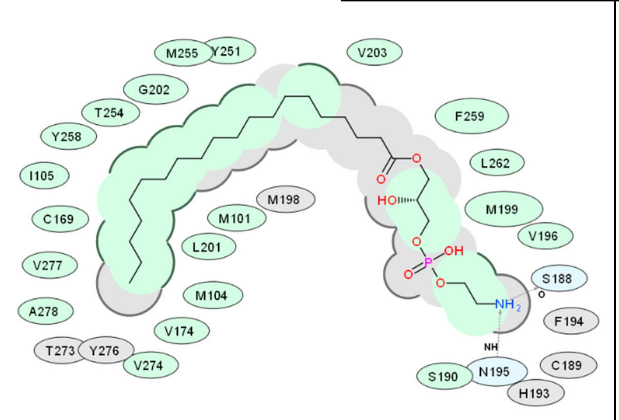

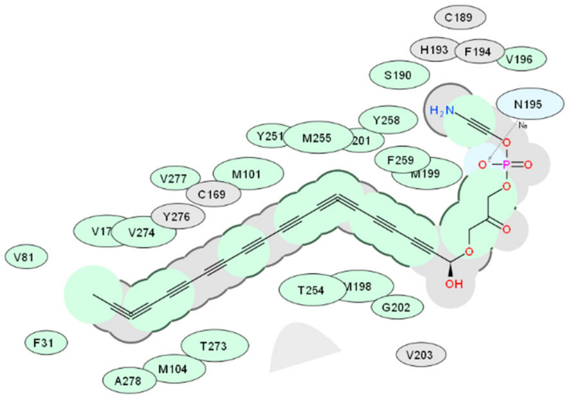

Myristyl sulfate (PubChem ID: 5248)

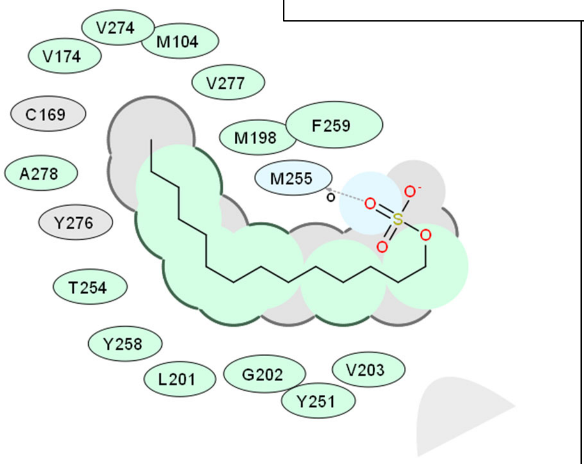

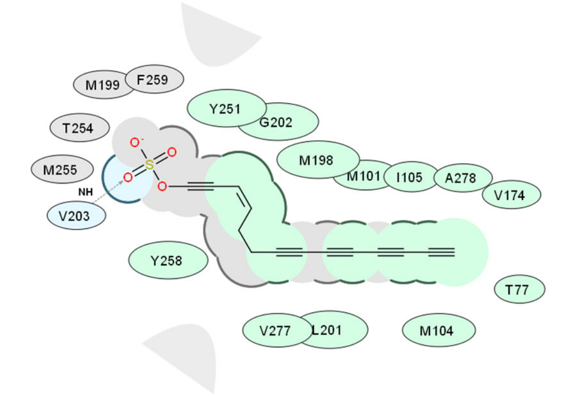

Figure S8: Interacting residues of the complexes with 1U19-based-*holo* model, before and after molecular dynamics. Hydrophobic regions in green, van der Waals interactions in gray surface accessible regions in gray parabolas; hydrogen bond acceptors in blue.

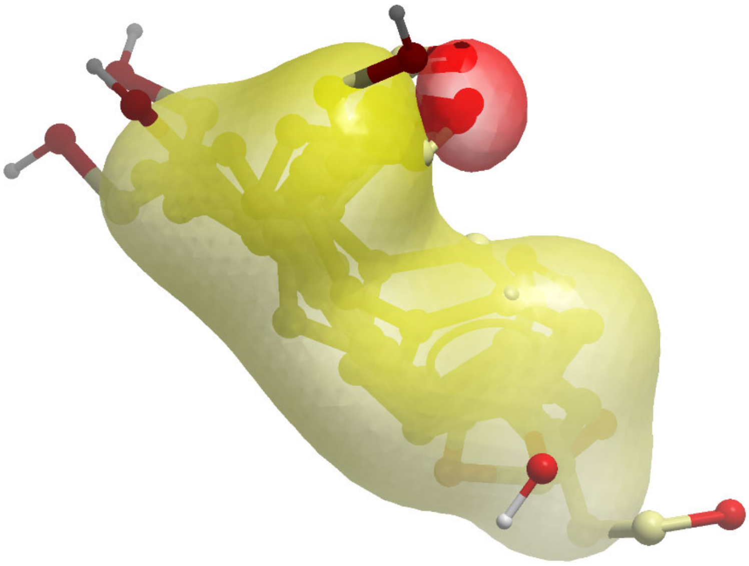

Figure S9: **APF superposition based pharmacophore:** Yellow mesh represents the lipophilic property while red mesh meant for charge property.

Table S7: Compounds retrieved after stage 1 screening

Pubchem_CID	APF score	Pubchem_CID	APF score	Pubchem_CID	APF score
202225	-149.3	15818	-121.7	246164	-121.7
15559396	-126.9	167918	-121.6	5284512	-121.7
440071	-124.4	53477683	-121.6	9929317	-118.9
53477695	-124.1	70678558	-121.6	53480452	-118.9
53477755	-123.7	2724385	-121.6	5880	-118.8
21116917	-123.5	441301	-121.5	65727	-118.8
126511	-123.5	24779614	-121.5	247732	-118.8
6675	-123.4	44263345	-121.4	53477687	-118.7
11740284	-123.4	22833601	-121.4	53477686	-118.7
9963687	-123.4	65555	-121.4	3034755	-118.7
221122	-123.3	246873	-121.4	5879	-118.6
12443252	-123.3	53480447	-121.4	50192	-118.5
221493	-123.3	228491	-121.4	159663	-118.5
222528	-123.3	94235	-121.3	5283632	-118.5
21252309	-123.3	23617285	-121.3	53477678	-118.4
21252318	-123.2	3531157	-121.3	101771	-118.4
123796	-123.2	92787	-121.2	65076	-118.4
3032822	-123	9863245	-121	53477894	-118.3
21125002	-122.9	439501	-120.9	53480457	-118.3
10253562	-122.8	134494	-120.8	114976	-118.3
12310288	-122.8	42622727	-120.7	644073	-118.3
164853	-122.7	21252312	-120.6	65728	-118.3
53477702	-122.6	188292	-120.5	53480451	-118.3
31401	-122.6	53477693	-120.5	92810	-118.3
122340	-122.6	22833539	-120.5	225688	-118.2
10133	-122.5	53477689	-120.5	53477807	-118.2
127601	-122.5	6665	-120.4	53480461	-118.2
160499	-122.5	22833540	-120.3	53480464	-118.2
53477753	-122.5	66066	-120.3	6011	-118.2
115245	-122.4	92786	-120.3	92997	-118.2
22833530	-122.4	53477756	-120.1	441289	-118.2
387316	-122.3	44725717	-120.1	18665256	-118.2
122312	-122.3	92748	-120	439577	-118.2
5283853	-122.2	53477752	-119.8	5281326	-118.2
5283956	-122.2	101790	-119.7	6436907	-118.2
164672	-122.1	68873	-119.6	4225619	-118.2
53477716	-122.1	91498	-119.6	53480460	-118.1
441207	-122.1	11055399	-119.4	271605	-118.1
2733768	-122.1	53480454	-119.4	53480458	-118.1
28620	-122.0	13919536	-119.3	53477889	-118.1
122166	-122.0	53480455	-119.3	53480463	-118.1
222284	-121.8	22833587	-119.3	91477	-118.1
3032833	-121.8	39765	-119.2	53477890	-118.0

Pubchem_CID	APF score	Pubchem_CID	APF score	Pubchem_CID	APF score
53477793	-118.0	270604	-116.6	21775588	-113.8
3000226	-118	6452574	-116.6	5881	-113.8
441290	-119.2	5283637	-118	443078	-116.5
242332	-119.1	44263365	-118	20845972	-116.5
5280794	-118	656583	-116.5	102030	-113.7
64945	-118	6166	-116.5	6230	-113.6
11025495	-118	443872	-116.4	5282181	-113.6
53480449	-118	53477696	-116.3	9051	-113.5
92747	-118	105074	-116.3	12594	-113.5
5287939	-118	114833	-116.3	13109	-113.2
53480462	-117.9	5994	-116.3	5745	-113.1
13828666	-117.9	128251	-116.2	3080587	-113.1
53477891	-117.9	108192	-116.1	5280795	-113.1
12444617	-117.9	65543	-116.0	21700	-112.9
405	-117.9	5753	-116.0	222786	-112.7
99486	-117.9	247839	-115.9	5283731	-112.6
123976	-117.9	6013	-115.8	5280453	-112.6
246520	-117.9	10634	-115.8	5460852	-112.4
53477893	-117.8	15209	-115.7	6918296	-112.4
53477794	-117.8	14009228	-115.7	25200718	-112.3
53480459	-117.8	13789	-115.7	101770	-112.3
121948	-117.8	5995	-115.5	129846	-112.3
53480456	-117.8	119207	-115.5	5311067	-112.2
53477892	-117.8	160706	-115.5	5311051	-112.1
6010	-117.8	44263342	-115.5	53480448	-111.7
123743	-117.8	40973	-115.4	12895043	-111.5
6479222	-117.8	440707	-115.4	5875	-111.5
10286365	-117.7	6238	-115.2	470824	-111.5
53477732	-117.6	84088	-115.2	5311052	-111.4
5878	-117.6	5284537	-115.2	23724604	-111.4
42621273	-117.6	5754	-115.1	39507	-111.2
3247060	-117.6	9818021	-115.1	5743	-111
5283660	-117.6	10204	-115.1	6741	-110.8
3081084	-117.5	91451	-115.1	443980	-110.8
473141	-117.4	13783449	-114.9	71415	-110.6
5833	-117.4	44263343	-114.9	5311000	-110.6
25249	-117.4	11222	-114.9	24758425	-110.6
10635	-117.3	222803	-114.8	44263344	-110.5
537186	-117.1	6540478	-114.7	9782	-110.5
10494	-116.9	152971	-114.6	128210	-110.5
247020	-116.8	4746	-114.3	441335	-110.5
222865	-116.8	25201885	-114.2	6128	-110.4
6446	-116.7	9270	-114.1	82153	-110.4
101850	-116.7	246983	-114.0	443936	-110.4
18526330	-116.61	31378	-113.9	444036	-110.4

Pubchem_CID	APF score	Pubchem_CID	APF score	Pubchem_CID	APF score
13752005	-110.4	5280845	-106.1	9547243	-101.4
444033	-110.3	9854073	-106.0	5282381	-101.4
235905	-113.8	6215	-110.2	5281104	-105.8
439423	-113.8	14986	-110.2	5283043	-105.8
252379	-110	3033968	-105.7	36314	-101.3
53477727	-109.9	92094	-105.7	52923675	-101.3
4240	-109.9	102861	-105.6	24778711	-101.2
443958	-109.8	22833566	-105.5	537294	-101.2
9642	-109.7	68929	-105.4	24778618	-101.2
267302	-109.7	6444033	-105.4	1563	-101.2
5865	-109.6	9046	-105.2	53480922	-101.1
107873	-109.6	9547634	-105.2	7800	-101.1
17756771	-109.5	123929	-105	75364	-101.1
324224	-109.4	2826718	-104.9	123409	-101.0
20848950	-109.3	10380830	-104.7	53478669	-101.0
6857447	-109.2	6291	-104.7	5311221	-101.0
9878	-109.0	6438156	-104.7	25311	-101.0
9865442	-109.0	545608	-104.5	53479175	-100.9
26840	-109.0	5991	-104.5	10957631	-100.9
9972843	-108.9	10417998	-104.5	20848956	-100.9
48175	-108.9	11243969	-104.4	3034010	-100.9
5281004	-108.9	5280936	-104.4	537297	-100.8
5755	-108.7	60198	-104.0	53480486	-100.8
31307	-108.7	44263360	-103.8	11005	-100.8
5311066	-108.6	441080	-103.7	24778719	-100.8
6917715	-108.1	5280939	-103.6	52923467	-100.8
2117	-108.1	22207	-103.5	10459	-100.8
52421	-108.0	6918462	-103.2	24779527	-100.8
5280435	-107.9	53232	-103.0	53478185	-100.7
14985	-107.8	86609	-102.9	52924408	-100.7
229455	-107.4	54454	-102.7	53479131	-100.7
6918140	-107.4	5361092	-102.5	55245	-100.7
301115	-107.4	656719	-102.4	6443157	-100.7
9677	-107.3	446596	-102.3	20057319	-100.7
161273	-106.8	53477772	-102.2	27902	-100.7
5283118	-106.8	12918533	-102.2	214	-100.7
3042	-106.5	17835	-102.2	3084463	-100.7
5284607	-106.5	8207	-101.9	53478457	-100.6
9904	-106.5	5248	-101.8	985	-100.6
148124	-106.3	68911	-101.8	53477952	-100.5
6443809	-106.2	19800	-101.8	53480501	-100.5
5280793	-106.2	9939965	-101.7	52923059	-100.5
9547260	-106.1	5462340	-101.7	13849	-100.5
5311071	-106.1	53479181	-101.5	6438186	-100.5
5280483	-106.1	6431015	-101.5	53478086	-100.4

Pubchem_CID	APF score
53477961	-100.5
9544195	-100.5
5288783	-101.4
53478267	-101.3
3082163	-100.4
8209	-100.4
53479281	-100.4
52926307	-100.4
53478188	-100.4
53480503	-100.4
52924728	-100.3
46879024	-100.3
53480499	-100.3
9544087	-100.3
53477971	-100.3
53477967	-100.3
52923942	-100.3
52925131	-100.3
53478238	-100.2
53477966	-100.2
52924213	-100.2
52922901	-100.2
53480997	-100.2
53478057	-100.1
13734178	-100.1
9546843	-100.1
9547094	-100.1
53480972	-100.1
53478617	-100.1
6918289	-100.1
5280723	-100.1
9543815	-100.1
24779479	-100.1
53479727	-100.0
53480827	-100.0
9544681	-100.0
53478027	-100.0
53478051	-100.0
53480729	-100.0

Table S8: Docking scores for known ligands of OR1A2

Ligand	ICM docking score
(-)-carveol	-20.783
(*S*)-(-)-citronellal	-13.6861
(*R*)-(+)-citronellol	-13.1332
Citronellol	-17.0386
4-decenal	-14.9842
Geranial	-17.2807
Geraniol	-24.0937
Helional	-13.9991
Heptanal	-15.6382
Hydroxy-citronellal	-16.1748
Nonanal	-11.8318
Octanal	-12.7552
Octanol	-12.9535

TableS9: Metabolites retrieved after Stage-2 scanning with APF and docking scores: metabolites selected for molecular dynamics are bold.

Pubchem_CID	ICM Docking Score	Compound name	APF score
**22207**	**-14.49**	**14-methylhexadecanoic acid**	**-103.5**
**13849**	**-14.45**	**Pentadecanoic acid**	**-100.5**
**10459**	**-10.96**	**Hexadecanedioic acid**	**-100.8**
**1563**	**-10.87**	**2-hydroxytetradecanoic acid**	**-101.2**
**985**	**-10.44**	**Palmitic acid**	**-100.6**
60198	-9.032	Exemestane	-104
17835	-8.278	4-methyldecane	-102.2
20848956	-8.11	3-hydroxytetradecanedioic acid	-100.9
102030	-7.921	16-hydroxydehydroepiandrosterone	-113.7
7800	-7.728	Ethyl dodecanoate	-101.1
11005	-7.44	Tetradecanoic acid	-100.8
301115	-7.135	1,1-dicyclopentylethane	-107.4
247839	-6.545	Medrysone	-115.9
324224	-6.526	Beta-Bourbonene	-109.4
46879024	-6.525	1-pentadecanoyl-glycerol	-100.3
86609	-5.901	Alpha-Cubebene	-102.9
9051	-5.767	Dydrogesterone	-113.5
119207	-5.687	Testosterone sulfate	-115.5
102861	-5.432	Methyl dihydrojasmonate	-105.6
6013	-5.397	Testosterone	-115.8

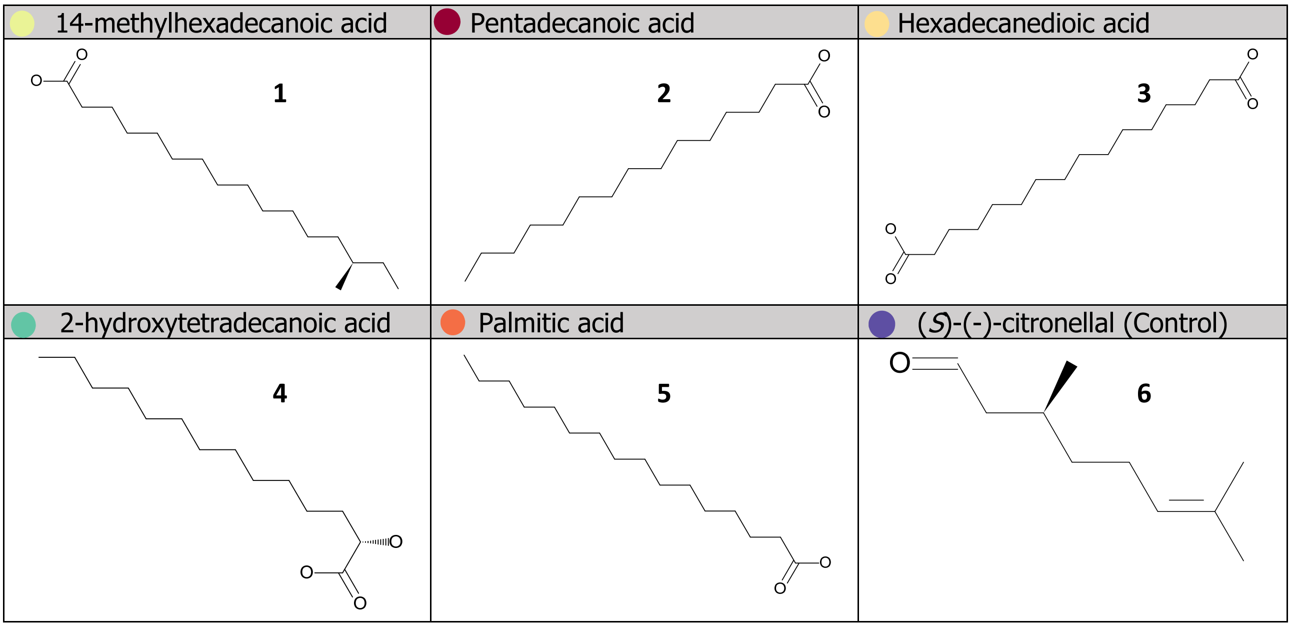

Figure S10: Chemical structures of the top five metabolites (1-5) selected for molecular dynamics, along with the control (**6**).

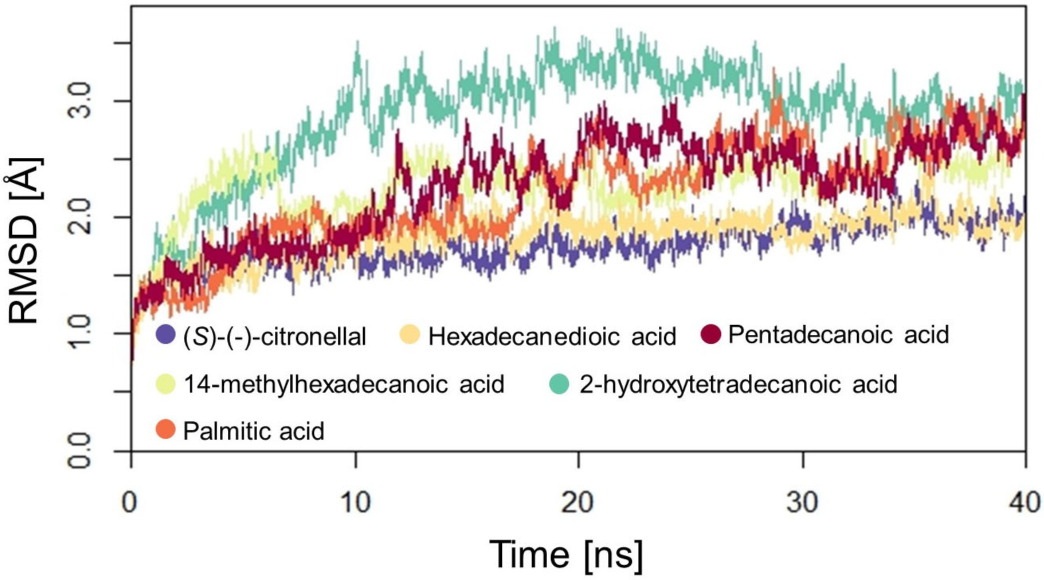

Figure S11: RMSD (Cα atoms) for control ((*S*)- (-)-citronellal) and predicted complexes.

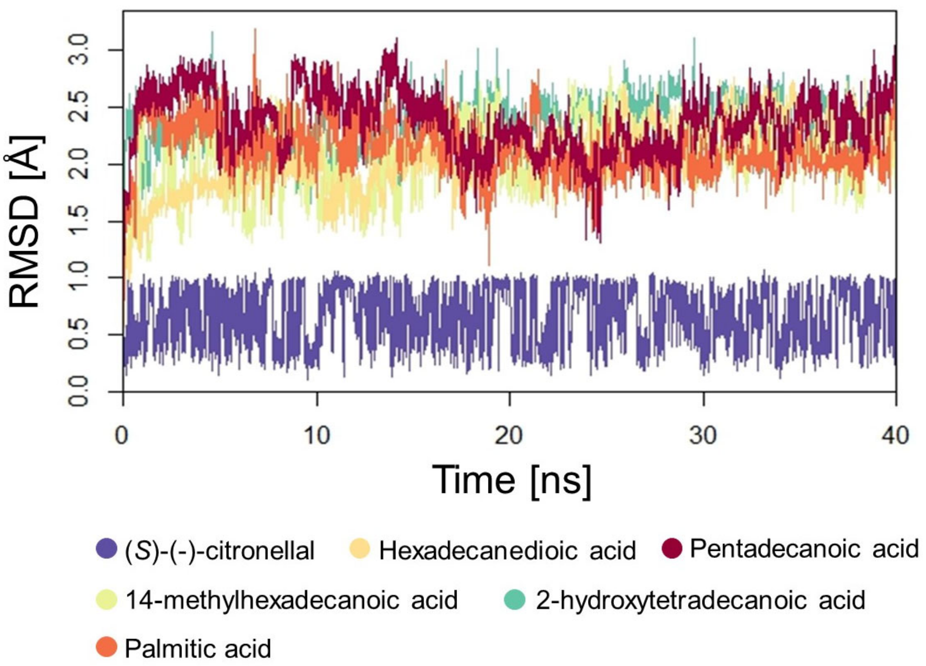

Figure S12: RMSD of the control ((*S*)- (-)-citronellal) and predicted ligands for OR1A2

<table>
<tr><th>Interacting residues and type of interaction before MD production</th><th>Interacting residues and type of interaction after MD production (40ns)</th></tr>
<tr><td colspan="2">OR1A2- (S)-(-)-citronellal (6: control)</td></tr>
<tr><td>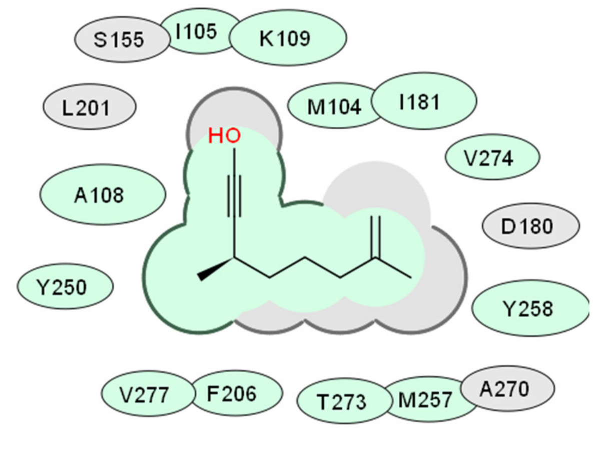
</td><td>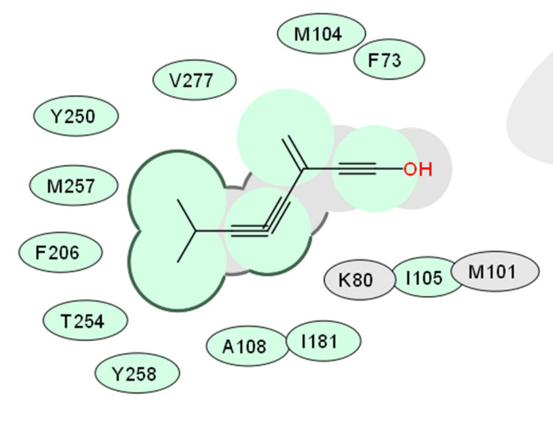
</td></tr>
<tr><td colspan="2">OR1A2- Pentadecanoic acid (predicted 1)</td></tr>
<tr><td>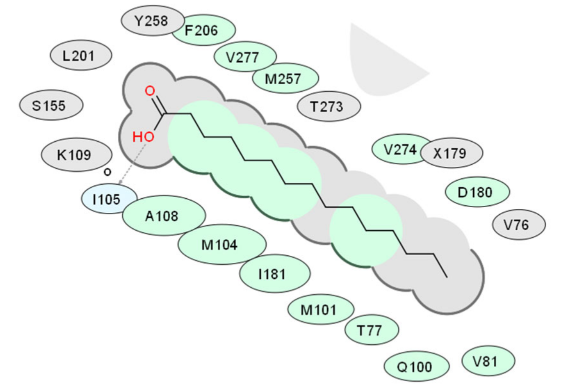
</td><td>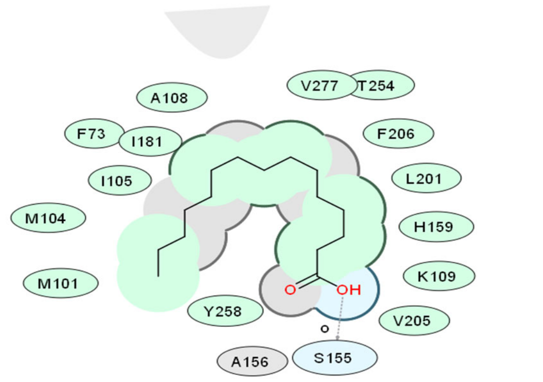
</td></tr>
<tr><td colspan="2">OR1A2- Hexadecanedioic acid (predicted 2)</td></tr>
<tr><td>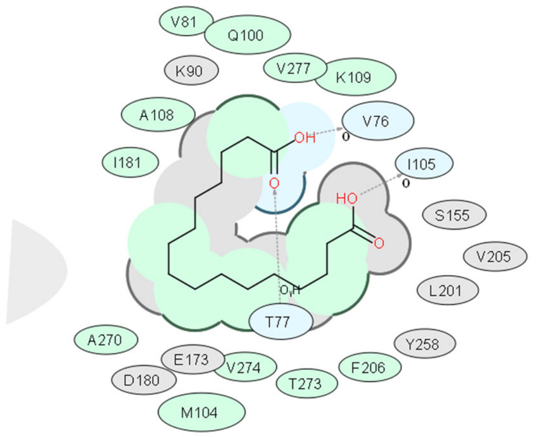
</td><td>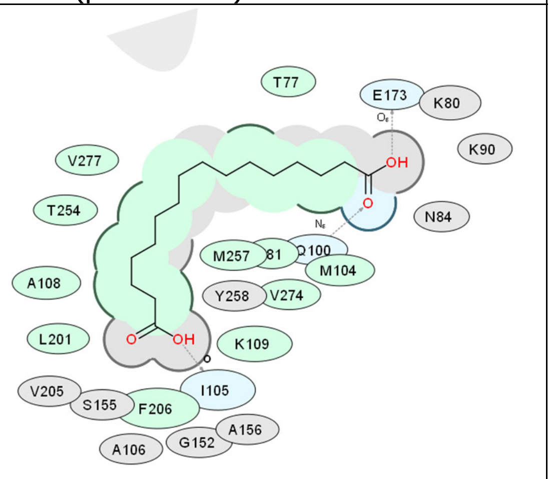
</td></tr>
</table>

Interacting residues and type of interaction before MD simulation	Interacting residues and type of interaction after MD simulation (40ns)
OR1A2- 14-methylhexadecanoic acid (predicted **3**)	
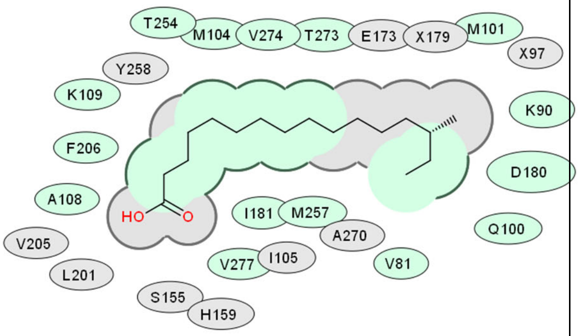	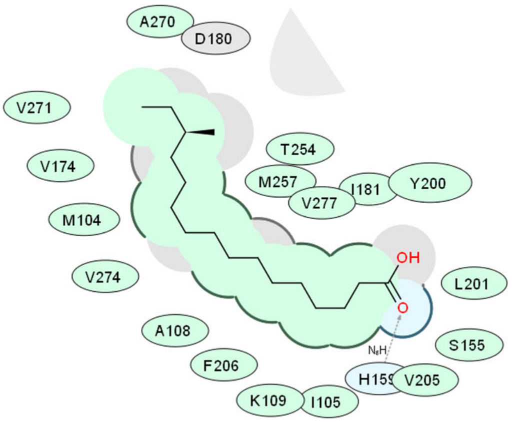
OR1A2-2-hydroxytetradecanoic acid (predicted **4**)	
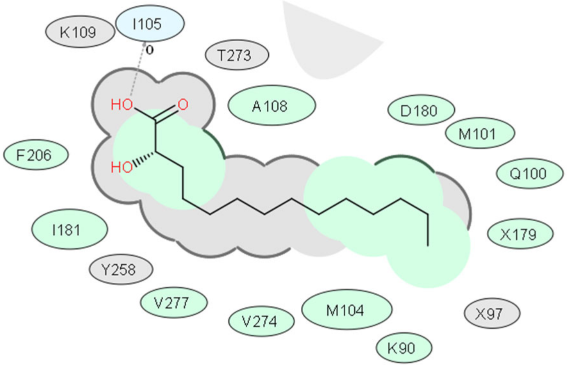	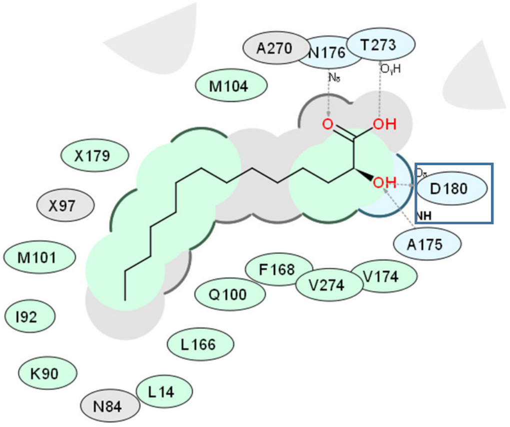
OR1A2- Palmitic acid (predicted **5**)	
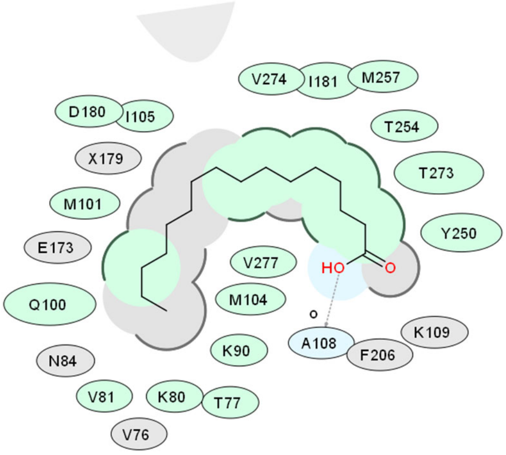	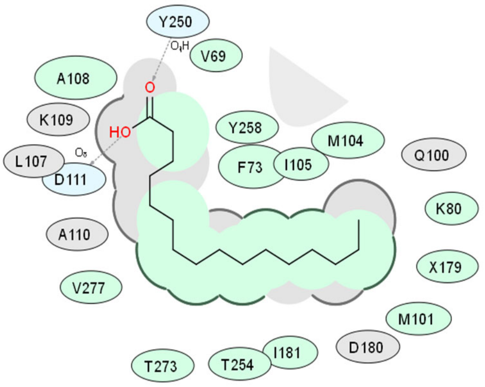

Figure S13: Interacting residues of the complexes before and after molecular dynamics. Hydrophobic regions in green, van der Waals interactions in gray surface accessible regions in gray parabolas; hydrogen bond acceptors in blue and cysteine residues labelled X.

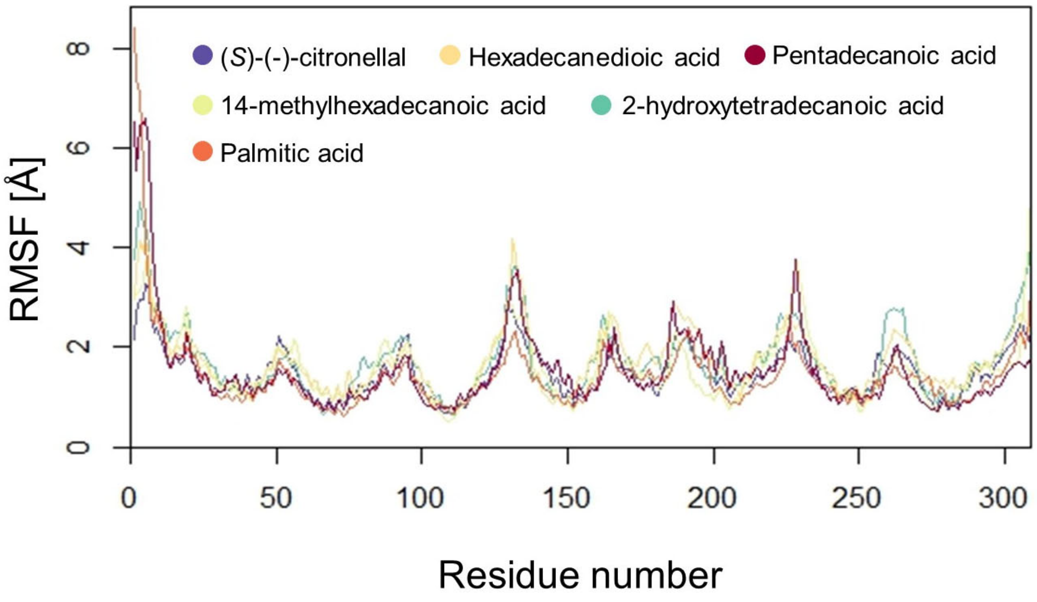

Figure S14: RMSF of the control ((*S*)- (-)-citronellal) and predicted ligands for OR1A2

References:

1. Schmiedeberg, K., et al., *Structural determinants of odorant recognition by the human olfactory receptors OR1A1 and OR1A2.* J Struct Biol, 2007. **159**(3): p. 400-12.
2. Massberg, D., et al., *Monoterpene (-)-citronellal affects hepatocarcinoma cell signaling via an olfactory receptor.* Arch Biochem Biophys, 2015. **566**: p. 100-9.
